# Maternal hyperglycemia induces alterations in hepatic amino acid, glucose and lipid metabolism of neonatal offspring: Multi-omics insights from a diabetic pig model

**DOI:** 10.1016/j.molmet.2023.101768

**Published:** 2023-07-04

**Authors:** Bachuki Shashikadze, Libera Valla, Salvo Danilo Lombardo, Cornelia Prehn, Mark Haid, Fabien Riols, Jan Bernd Stöckl, Radwa Elkhateib, Simone Renner, Birgit Rathkolb, Jörg Menche, Martin Hrabĕ de Angelis, Eckhard Wolf, Elisabeth Kemter, Thomas Fröhlich

**Affiliations:** 1 Laboratory for Functional Genome Analysis (LAFUGA), Gene Center, LMU Munich, 81377 Munich, Germany; 2 Molecular Animal Breeding and Biotechnology, Gene Center and Department of Veterinary Sciences, LMU Munich, 81377 Munich, Germany; 3 MWM Biomodels GmbH, 84184 Tiefenbach, Germany; 4 Max Perutz Labs, Vienna Biocenter Campus (VBC), 1030 Vienna, Austria; 5 University of Vienna, Center for Molecular Biology, Department of Structural and Computational Biology, 1030 Vienna, Austria; 6 CeMM Research Center for Molecular Medicine of the Austrian Academy of Sciences, 1090 Vienna, Austria; 7 Metabolomics and Proteomics Core (MPC), Helmholtz Zentrum München, 85764 Neuherberg, Germany; 8 German Center for Diabetes Research (DZD), 85764 Neuherberg, Germany; 9 Center for Innovative Medical Models (CiMM), LMU Munich, 85764 Oberschleißheim, Germany; 10 Institute of Experimental Genetics, German Mouse Clinic, Helmholtz Munich, 85764 Neuherberg, Germany; 11 University of Vienna, Faculty of Mathematics, 1090 Vienna, Austria; 12 Experimental Genetics, School of Life Science Weihenstephan, Technische Universität München, 85354 Freising, Germany

**Keywords:** Maternal diabetes, Neonates, Liver, Pig model, Proteomics, Metabolomics, Protein–protein interaction, Clinical parameters

## Abstract

**Objective:**

To gain mechanistic insights into adverse effects of maternal hyperglycemia on the liver of neonates, we performed a multi-omics analysis of liver tissue from piglets developed in genetically diabetic (mutant *INS* gene induced diabetes of youth; MIDY) or wild-type (WT) pigs.

**Methods:**

Proteome, metabolome and lipidome profiles of liver and clinical parameters of serum samples from 3-day-old WT piglets (n = 9) born to MIDY mothers (PHG) were compared with those of WT piglets (n = 10) born to normoglycemic mothers (PNG). Furthermore, protein–protein interaction network analysis was used to reveal highly interacting proteins that participate in the same molecular mechanisms and to relate these mechanisms with human pathology.

**Results:**

Hepatocytes of PHG displayed pronounced lipid droplet accumulation, although the abundances of central lipogenic enzymes such as fatty acid-synthase (FASN) were decreased. Additionally, circulating triglyceride (TG) levels were reduced as a trend. Serum levels of non-esterified free fatty acids (NEFA) were elevated in PHG, potentially stimulating hepatic gluconeogenesis. This is supported by elevated hepatic phosphoenolpyruvate carboxykinase (PCK1) and circulating alanine transaminase (ALT) levels. Even though targeted metabolomics showed strongly elevated phosphatidylcholine (PC) levels, the abundances of multiple key enzymes involved in major PC synthesis pathways – most prominently those from the Kennedy pathway – were paradoxically reduced in PHG liver. Conversely, enzymes involved in PC excretion and breakdown such as PC-specific translocase ATP-binding cassette 4 (ABCB4) and phospholipase A2 were increased in abundance.

**Conclusions:**

Our study indicates that maternal hyperglycemia without confounding obesity induces profound molecular changes in the liver of neonatal offspring. In particular, we found evidence for stimulated gluconeogenesis and hepatic lipid accumulation independent of *de novo* lipogenesis. Reduced levels of PC biosynthesis enzymes and increased levels of proteins involved in PC translocation or breakdown may represent counter-regulatory mechanisms to maternally elevated PC levels. Our comprehensive multi-omics dataset provides a valuable resource for future meta-analysis studies focusing on liver metabolism in newborns from diabetic mothers.

## Highlights

• Maternal hyperglycemia without confounding obesity induces profound molecular changes in the liver of neonatal offspring.
• Increased hepatic phosphoenolpyruvate carboxykinase and serum free fatty acid levels argue for stimulated gluconeogenesis.
• Prominent lipid accumulation in hepatocytes is observed despite downregulation of enzymes involved in lipogenesis.
• Reduced lipogenesis in tandem with an increased lipid breakdown may counteract maternally elevated lipid levels.

## Graphical abstract

**Figure:**
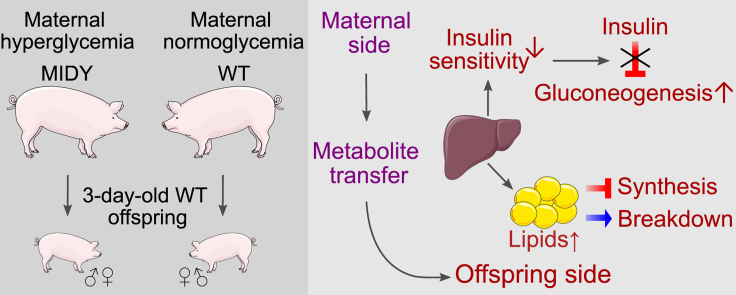

## Abbreviations

AGC: automatic gain control
BGC: blood glucose concentration
BP: biological process
CC: cellular component
CIA: co-inertia analysis
DIA: data independent acquisition
DNL: *de novo* lipogenesis
GDA: gene-disease association
GDM: gestational diabetes mellitus
GO: gene ontology
GPF: gas phase fractionation
GWAS: genome-wide association studies
HOMA-IR: homeostatic model assessment for insulin resistance
KEGG: Kyoto Encyclopedia of Genes and Genomes
LC-MS/MS: nano-liquid chromatography–tandem mass spectrometry analysis
LOOCV: leave-one-out cross-validation
MF: molecular function
MIDY: mutant *INS* gene induced diabetes of youth
NAFLD: non-alcoholic fatty liver disease
NCE: normalized collision energy
NEFA: non-esterified free fatty acids
OPLS-DA: orthogonal projection to latent structures discriminant analysis
ORA: over-representation analysis
PC: phosphatidylcholine
PCA: principal component analysis
PE: phosphatidylethanolamine
PHG: wild-type piglet born to transgenic hyperglycemic pig
PNG: wild-type piglet born to normoglycemic pig
PPI: protein-protein interaction network
QUICKI: quantitative insulin sensitivity check index
SIDD: severe insulin deficient diabetes
SM: sphingolipid
TG: triglyceride
VIP: variance importance in projection
WT: wild-type

## 1 Introduction

A disturbed prenatal environment is considered a risk factor for health complications in offspring [1]. The environment of the developing fetus is influenced by an altered maternal nutritional and metabolic state [2,3]. For example, *in utero* exposure to elevated maternal glucose can trigger long-term consequences in the physiology and metabolism of the offspring [4]. Offspring of mothers with gestational diabetes mellitus (GDM) have a fourfold increased risk of developing a metabolic syndrome [5]. So far, hyperglycemia-related fetal programming has been mainly investigated by epidemiological studies and reports at the molecular level are scarce. Furthermore, human patient data have the drawback that confounding factors, such as the mother's lifestyle and medical history, are frequently not completely recorded. On the other hand, animal models living in controlled laboratory conditions with standardized tissue sampling [6] allow differentiating the sole consequences of maternal hyperglycemia from those of comorbidities. The pig is a promising large animal model to fill the gap between proof-of-concept studies and clinical trials [[7], [8], [9]]. In the context of diabetes and pregnancy, it is worth mentioning that pig offspring, similar to human babies, are born in a more mature state compared to rodent pups and are therefore also exposed to maternal glycemia in a later developmental phase [10]. Furthermore, piglets show similarities to human physiology in terms of changes in energy metabolism during both normal and pathological birth (reviewed in [11]). Since the liver is responsible for maintaining normal blood glucose levels alongside the homeostasis of other relevant metabolites such as lipids and amino acids [12], it is especially relevant for the consequences of maternal diabetes on offspring. Furthermore, as a major metabolic organ, the liver is highly relevant in the context of metabolic syndromes. Interestingly, previous studies of both human cohorts and rodent models suggest that maternal diabetes may be associated with offspring markers of liver pathology mainly related to an aberrant lipid metabolism [[13], [14], [15], [16], [17]]. The involvement of metabolic organs in neonatal complications is further suggested by the study of Renner et al. where it was found that maternal hyperglycemia, even in the absence of maternal and neonatal obesity, was associated with alterations in the neonatal offspring's plasma metabolome (such as amino acids and lipids) [18]. The detection of molecular changes induced by prenatal exposure to maternal hyperglycemia and underlying biological pathways could provide the basis for novel intervention strategies which could have far-reaching implications for child health care. In this study, we address hepatic proteome and metabolome alterations alongside clinical-chemical changes and histomorphological findings in piglets developed in genetically hyperglycemic *INS*^C94Y^ transgenic pigs, a model for mutant *INS* gene induced diabetes of youth (MIDY) [19].

## 2 Materials and methods

### 2.1 Biological samples

In this study, the hepatic proteome, metabolome as well as clinical-chemical parameters in serum from 3-day-old wild-type (WT) piglets born to hyperglycemic mothers (PHG) expressing the mutant insulin C94Y [19] were compared to the profiles of WT piglets born to normoglycemic mothers (PNG). To further complement molecular findings histomorphological evaluation of the liver was performed. The hyperglycemic *INS*^C94Y^ transgenic pig model was obtained using the *INS*^C94Y^ expression vector, including the porcine *INS* gene with a point mutation introducing a Cys→Tyr exchange in position 94, which disrupts one of the two disulfide bonds between the A- and B-chain of the mature insulin molecule. This generates a misfolded insulin protein that induces endoplasmic reticulum stress in the β-cells, resulting in early-onset permanent insulin-deficient diabetes mellitus and β-cell loss [19]. The non-diabetic and diabetic sows used in this project were half-siblings produced by mating a diabetic boar with different WT sows. The piglets of the PHG and PNG groups were derived from mating of non-diabetic and diabetic sows with the same WT boar, reducing genetic variance. The diabetic sows were treated daily with a combination of long-acting and short-acting insulin, to ensure a blood glucose concentration (BGC) in a physiological range (around 150 mg/dL). This physiological range was maintained in the diabetic sows also during the mating and during the first 3 weeks of pregnancy to ensure the pregnancy state. After this period, the amount of insulin administrated was reduced, to obtain a BGC of around 300 mg/dL, which corresponds to a pathological diabetic situation during pregnancy. 30 min after birth and before first milk intake, blood glucose of newborn piglets was measured by ear vein puncture using a glucometer (FreeStyle-Freedom Lite). In addition, venous EDTA plasma samples of offspring were collected, stored at −80 °C for determination of insulin concentration. Sows were housed in groups under control conditions, with free access to water and fed with commercial food once per day. Shortly before giving birth, sows were separated into a separate pen in the farrowing unit. Newborn piglets were housed in the farrowing pen together with the mother, and a heated nest was offered to the piglets. At an age of 3 days, non-fasted piglets underwent necropsy. Tissues were collected by random systematic sampling [6], shock-frozen on dry ice and stored at −80 °C until analysis. For omics analyses, all samples were processed in parallel to avoid possible bias related to different storage times. All experiments were performed according to the German Animal Welfare Act (Deutsches Tierschutzgesetz), following the ARRIVE guidelines and Directive 2010/63/EU.

### 2.2 Proteomics

#### 2.2.1 Sample preparation

Frozen liver tissue samples were transferred into prechilled tubes and cryo-pulverized in a CP02 Automated Dry Pulverizer (Covaris, Woburn, MA, USA) using an impact level of 3 according to the manufacturer's instructions. Powdered tissue was lysed in 8 M urea/0.5 M ammonium bicarbonate (Roche Diagnostics, Mannheim, Germany) by ultrasonication (18 cycles of 10 s) using a Sonopuls HD3200 (Bandelin, Berlin, Germany). Pierce 660 nm Protein Assay (Thermo Fisher Scientific, Rockford, IL, USA) was used for protein quantification. 20 μL of lysate containing 20 μg of protein were processed for digestion. Disulfide bonds were reduced with 45 mM dithiothreitol/20 mM tris(2-carboxyethyl) phosphine (30 min, 56 °C). Reduced cysteine side chains were alkylated by adding 100 mM iodoacetamide (30 min, room temperature), followed by quenching the remaining iodoacetamide with dithiothreitol (90 mM, 15 min, room temperature). Sequential 2-step digestion was performed, firstly with Lys-C (FUJIFILM Wako Chemicals Europe GmbH, Neuss, Germany) for 4 h (1:50 enzyme to protein ratio) and subsequently with modified porcine trypsin (Promega, Madison, WI, USA) for 16 h at 37 °C (1:50 enzyme to protein ratio). After digestion, samples were dried before analysis using a vacuum centrifuge.

#### 2.2.2 Nano-liquid chromatography–tandem mass spectrometry analysis

Nano-liquid chromatography–tandem mass spectrometry (LC-MS/MS) analysis was performed on an UltiMate 3000 nano-LC system coupled to a Q Exactive HF-X Orbitrap mass spectrometer via a nano-electrospray ion source (all Thermo Fisher Scientific). 1 μg of peptides were transferred to a PepMap 100 C18 trap column (100 μm × 2 cm, 5 μM particles, Thermo Fisher Scientific) and separated on an analytical column (PepMap RSLC C18, 75 μm × 50 cm, 2 μm particles, Thermo Fisher Scientific) at 250 nL/min with an 80-min gradient of 5–20% of solvent B followed by a 9-min increase to 40%. After the gradient, the column was washed with 85% solvent B for 9 min, followed by 10-min re-equilibration with 3% solvent B. Mobile phases A and B were 99.9/0.1% water/formic acid (v/v) and 99.9/0.1% acetonitrile/formic acid (v/v), respectively. Gas phase fractionation (GPF)-based chromatogram libraries [20] were built using 6 injections of pooled samples with 25 × 4 m/z-wide data-independent acquisition (DIA) (30,000 resolution, AGC target 1e6 maximum inject time 55 ms, NCE 27, +3H assumed charge state) spectra using a staggered window pattern with window placements optimized by Skyline (v.22.2) (i.e. 400.43–502.48, 500.48–602.52, 600.52–702.57, 700.57–802.61, 800.61–902.66, 900.66–1002.70), yielding 300 × 2 m/z-wide windows spanning from 400 to 1000 *m*/*z* after deconvolution. For DIA measurements, 50 × 12 m/z-wide (in the range of 400–1000 *m*/*z*) precursor isolation window DIA spectra (15,000 resolution, AGC target 1e6, maximum inject time 20 ms, NCE 27) was acquired as described in [21] using a staggered window pattern [22]. Window placements were calculated by Skyline software [23]. Precursor spectra (in the range of 390–1010 *m*/*z*, 60,000 resolution, AGC target 1e6, max IIT 60 ms, +3H assumed charge state) were interspersed every 50 MS/MS spectra.

#### 2.2.3 Peptide and protein identification and quantification

Protein intensities were extracted from the DIA data using predicted spectral libraries generated by DIA-NN's (v1.8.1) built-in deep-learning-based spectra and retention time predictor which was further refined by the experimental data from project-specific GPF-based library (also generated by DIA-NN). For this, the *Sus scrofa* protein database (UniProt Reference Proteome – Taxonomy 9823 – Proteome ID UP000008227, 49,792 entries) alongside the MaxQuant contaminants fasta file [24] were used. Only tryptic peptides with a maximum of one missed cleavage and charge state of +2, +3 and +4 were considered. Cysteine carbamidomethylation was selected as a fixed modification and the quantification strategy was set to robust LC (high precision mode). Retention time correction was performed automatically by DIA-NN and quantification strategy was set to Robust LC (high accuracy mode). Similarly, mass tolerance was determined automatically by DIA-NN and was set to 9 ppm and 18 ppm for MS1 and MS2, respectively. The “Genes” column was used to count unique proteins. All other settings were left default. DIA-NN's main output containing precursor level data was used for the downstream analysis in R using custom scripts. Briefly, the output was filtered at 1% false-discovery rate, using both global and run-specific q-values for precursors and global q-values for protein groups. Peptides derived from potential contaminants, non-proteotypic peptides and peptides with a low signal quality were removed. Precursor intensities for different charge states were summed to derive peptide intensities. Normalization of raw intensities was performed using the MaxLFQ algorithm [25]. Proteins detected in at least 60% of all replicates were kept for quantitative analysis. To handle missing values, data imputation was performed using a random forest algorithm with the R package *MissForest* [26].

#### 2.2.4 Western blot quantification

Powdered liver tissue was lysed in Laemmli extraction buffer supplemented with protease and phosphatase inhibitors (Complete®, Sigma-Aldrich) and protein concentration was determined by BCA assay. Equal amount of denatured tissue lysate per lane was separated on SDS-polyacrylamide minigels and blotted on PVDF membranes. Equal loading was controlled by Ponceau staining. The following primary antibodies were used: rabbit polyclonal antibody against ALDH1L2 (no. 21391-1-AP, dilution 1:4000, proteintech), rabbit polyclonal antibody against claudin 15 (no. 38-9200, dilution 1:1000, Thermo Scientific), rabbit polyclonal antibody against RAB3D (no. 12320-1-AP, dilution 1:1500, proteintech), and mouse monoclonal antibody against pan-actin (no. MAB1501, dilution 1:40,000; Sigma Aldrich). As secondary antibodies, HRP-labeled goat polyclonal antibody against rabbit IgG (no. 7074, dilution 1:2,000, Cell Signaling) and HRP-labeled goat polyclonal antibody against mouse IgG (no. 115-035-146, dilution 1:10,000, Jackson ImmunoResearch), respectively, were used. Bound antibodies were visualized using SuperSignal™ ECL reagents (Thermo Fisher Scientific) and ECL ChemoStar Imager (INTAS). Stripping was done to analyze ratio of various protein abundances and the reference protein. Therefore, membranes were incubated with the stripping buffer (2% SDS, 62.5 mm Tris/HCl, pH 6.7, and 100 mM beta-mercaptoethanol) for 60 min at 70 °C. Afterward, membranes were washed, blocked, and incubated with the next primary antibody. Signal intensities were quantified using ImageQuant (GE Healthcare). Standardization of equal loading was referred to the signal intensities of pan-actin of the corresponding PVDF membrane. Data are shown as mean ± SD.

#### 2.2.5 STRING network construction and characteristics

The pig-specific and human-specific networks were downloaded from STRING database v11.5 (https://string-db.org/) [27]. This large database includes several sources of information grouped in 7 evidence channels: neighborhood, fusion, co-occurrence, co-expression, experiments, knowledge, and text-mining. Each of these sources reflects different information (i.e. computational prediction of protein proximity, protein expression, literature knowledge) and contributes to obtaining a combined score. This is a metric that considers the probability of different evidence channels and corrects for the probability of randomly observing an interaction between two proteins. In both pig and human, we took into consideration 4 possible networks: full, full with high confidence interactions (combined score>0.7), physical (direct interactions only), and physical with high confidence (direct interactions and combined score>0.7). Based on the network connectivity of the differentially abundant proteins, we decided to proceed with our analyses with the full network and high-confidence interactions, resulting in 15,360 nodes with 170,244 edges for pigs and 16,793 proteins with 251,982 edges in humans.

#### 2.2.6 Mapping of dysregulated proteins in the PPIs networks

For this aim, we selected those proteins with adjusted p-value ≤0.05 and fold-change ≥1.5 and mapped them on the pig- and human-specific protein–protein interactions (PPI). For each network, we calculated the percentage coverage and the network connectivity distinguishing between up-regulated, down-regulated, and total differentially abundant proteins (Supplementary Figs. 1A–D). Network connectivity was calculated by computing a z-score of the largest connected component for each group of proteins and comparing it against 10,000 randomly selected protein sets of the same size.

#### 2.2.7 Identification and biological characterization of dysregulated proteins core

We checked whether each connected component among the up-regulated and down-regulated proteins would be statistically significant in pigs and in humans. Once we extracted the main cores among the up- and down-regulated proteins, we identified expanded networks that would connect at least 90% of the up- and down-regulated proteins respectively, including their interacting proteins. For this purpose, we have used a random walk with restart algorithm, setting the restarting parameter, alpha, equal to 0.9, ensuring that the propagation would remain close to the original set of seed genes. We expanded the seed genes (up- and down-regulated proteins) until 90% would be connected. The biological characterization of the protein cores and the expanded networks was performed by enrichment analyses for the three main branches of the gene ontology (GO) [28] biological processes (BP), molecular functions (MF), and cellular components (CC), and for Kyoto Encyclopedia of Genes and Genomes (KEGG) pathway [29] using GSEAPY [30].

#### 2.2.8 Disease relationship

Disease gene associations were retrieved from DisGeNet [31] which represents the largest publicly available collections of genes and variants associated with human diseases, including expert-curated associations from genome-wide association studies (GWAS) catalogues, animal models and scientific literature. Depending on the accuracy of the type of information, each gene–disease association is attributed with a gene–disease association (GDA) score that ranges from 0 to 1. We selected associations with a GDA score >0.3, retrieving information for 11,099 diseases. The relationship between each set of differentially abundant proteins (s1) and set of disease proteins (s2) was then computed in two different ways: 1) by calculating their Jaccard index (intersection (s1,s2)/union(s1,s2)), and by network proximity of the two sets [32]. Network proximity computes the closest distance between two sets of proteins in a network and by comparing it against 10,000 random sets of similar topological features. In this way, we considered and corrected for interactome biases such as the heavy-tail degree distribution and the discretization of other common network distances like the shortest path.

### 2.3 Targeted metabolomics

Targeted metabolomics measurements were performed using liquid chromatography- and flow injection-electrospray ionization-tandem mass spectrometry (LC- and FIA-ESI-MS/MS) and the Absolute*IDQ*™ p180 Kit (BIOCRATES Life Sciences AG, Innsbruck, Austria). The assay allows simultaneous quantification of 188 metabolites. For the LC-part, compounds were identified and quantified based on scheduled multiple reaction monitoring measurements (sMRM), for the FIA-part on MRM. The complete assay procedures as well as the tissue extraction have been previously published [33]. In brief, tissue homogenates were always prepared freshly as follows: frozen porcine liver tissue samples were weighed into homogenization tubes with ceramic beads (1.4 mm). For metabolite extraction, to each 1 mg of frozen porcine liver tissue 3 μL of a cooled mixture (4 °C) of ethanol/phosphate buffer (85/15 v/v) were added. Tissue samples were homogenized using a Precellys24 homogenizer (PEQLAB Biotechnology GmbH, Germany) three times for 30 s at 5,500 rpm and −4 °C, with 30 s pause intervals to ensure constant temperature, followed by centrifugation at 10,000×*g* for 5 min. Subsequently, 10 μL of the supernatants were analyzed with the p180 assay. Data evaluation for quantification of metabolite concentrations and quality assessment were performed with the software MultiQuant 3.0.1 (SCIEX) and the Met*IDQ*™ software package, which is an integral part of the Absolute*IDQ*™ Kit. Metabolite concentrations were calculated using internal standards and reported as pmol/mg for wet tissue.

### 2.4 Shotgun lipidomics

All standards were purchased from Avanti Polar Lipids: Ultimate SplashOne (#330820), dFA 18:1 (#861809), dFA 20:4 (#861810), dCer d18:0/13:0 (#330726), Glu Cer(d18:1-d7/15:0) (#330729), dLacCer d18:1/15:0 (#330727), 15:0-18:1-d7-PA (#791642), EquiSPLASH (#330731).

#### 2.4.1 Lipidomic sample extraction

15 μL (equivalent to 5 mg) of the liver homogenates (see 2.3 for procedure) were transferred into 1.5-mL glass vials together with 85 μL of MilliQ water (H_2_O). For accurate quantification, 25 μL of a mix of 77 deuterated internal standards were then added to the samples (Ultimate SplashOne, dFA 18:1, dFA 20:4, dCer d18:0/13:0, Glu Cer(d18:1-d7/15:0), dLacCer d18:1/15:0, 15:0-18:1-d7-PA). For lipid extraction, 160 μL of methanol (MeOH, Optigrade, Thermofisher) and 575 μL methyl tert-butyl ether (MTBE) were added followed by incubation for 30 min on an orbital shaker DOS-10L (Neolabline, Heidelberg, Germany) at 300 rpm. For phase separation, 200 μL of H_2_O was added to each vial and were centrifuged at 5,000×*g* for 10 min at room temperature with a Sigma 4-5C centrifuge (Qiagen, Hilden, Germany). The upper (organic) phase was evaporated with nitrogen gas using a Barkey evaporator (Barkey, Leopoldshoehe, Germany). The aqueous phase was again extracted with 100 μL MeOH and 300 μL MTBE. After addition of 100 μL H_2_O, the samples were incubated for 5 min at room temperature at 300 rpm and then centrifuged for 10 min at 5,000×*g*. The organic phase was transferred into the respective vial from the first extraction step and evaporated to dryness with gaseous nitrogen. Samples were reconstituted in 275 μL running solvent (10 mM ammonium acetate in Dichloromethane:MeOH (50:50, v/v)) and 267 μL were subsequently transferred into new vials with insert. For quality control purposes (QC-pool samples), 10 μL of each study sample were pooled. 15 μL aliquots were created and extracted with the above-described procedure. Additionally, 3 blank samples consisting of 15 μL EtOH/phosphate buffer were prepared and extracted.

#### 2.4.2 Shotgun lipidomics measurements

The DMS-SLA shotgun lipidomics assay is based on the method published by Baolong Su et al. [34]. All samples were measured with a SCIEX Exion UHPLC-system coupled to a SCIEX QTRAP 6500+ mass spectrometer equipped with a SelexION differential ion mobility interface (SCIEX, Darmstadt, Germany) operated with Analyst 1.6.3. 75 μL of the re-dissolved sample were injected using the running solvent (10 mM ammonium acetate in Dichloromethane:MeOH (50:50, v/v)) at an isocratic flow rate of 8 μL/min. After 9 min the flowrate was ramped to 30 μL/min for 2 min to allow washing. Each sample was analyzed using multiple reaction monitoring (MRM) in two consecutive flow injection analysis (FIA) runs. In the first run, phosphatidylcholines (PC), phosphatidylethanolamines (PE), phosphatidylglycerols (PG), phosphatidylinositols (PI), phosphatidylserines (PS), and sphingomyelins (SM) were separated with the SelexION DMS cell using field asymmetric ion mobility mass spectrometry (FAIMS) prior to analysis in the Turbo Spray IonDrive source of the mass spectrometer. To enhance the separation of the lipid classes, 1-propanol was used as a chemical modifier. In the second run, cholesteryl esters (CE), ceramides (Cer d18:1), dihydroceramides (Cer d18:0), lactosylceramides (LacCER), hexosylceramides (HexCER), phosphatidic acid (PA), lysophosphatidylcholines (LPC), lysophosphatidylethanolamines (LPE), lysophosphatidylglycerols (LPG), lysophosphatidylinositols (LPI), lysophosphatidylserines (LPS), free fatty acids (FFA), diglycerides (DG), and triglycerides (TG) were measured with the DMS-cell switched off. Lipids were quantified with the Shotgun Lipidomics Assistant (SLA) software (v1.3) by calculating the area ratio between the analyte and the respective internal standard [34]. Lipid concentrations (nmol/g) were corrected for isobaric overlap with SLA. The mass spectrometer was operated with the following conditions: curtain gas 20 psi, ion source gas 1 14 psi, ion source gas 2 20 psi, Collision gas medium, temperature 150 °C, separation voltage +3500 V, ion spray voltage +4200 and +4500 V in ESI+ mode and −4400 and −3300 V in ESI− mode for run 01 and 02, respectively. Prior to each batch, the DMS cell was tuned, and the stability and sensitivity of the instrument was checked with the EquiSPLASH mixture.

#### 2.4.3 Lipidomics data processing

The shotgun lipidomics raw data set contained 1,204 individual lipid species. Data were subsequently pre-processed using R (version 4.2.1). To assure high data quality, a multi-step procedure was applied: in the first step of this quality control (QC) procedure, lipids with missing values in more than 35% in the pool samples were discarded from the data set (n = 136). In the second step, the group-specific missingness was evaluated i.e., whether a specific lipid is observed in only one of the biological groups. Lipids exhibiting a group-wise missingness of 50% in all groups were discarded from the data set (n = 7). Next, lipids with a coefficient of variation >25%, determined by the QC-pool samples, were removed from the data set (n = 22). The last quality control step comprised the calculation of the dispersion ratio (D-ratio) for each lipid [35]:

$$\frac{\sigma_{tech}}{\left(\sqrt{\sigma_{biol}^{2}+\sigma_{tech}^{2}}\right)}$$

where $\sigma_{tech}^{2}$ is the technical variance determined by the variance of the QC-pool samples and $\sigma_{biol}^{2}$ is the biological variance given by the variance of the biological samples within the study. We used a D-ratio threshold of 50%, as this implies that the technical variance is higher than the biological variance (n = 43 lipids were removed). After quality control, 996 lipid species remained in the liver data set, which contained 445 missing values (equivalent to 2% of the data set). Missing values were imputed using the k-nearest-neighbor obs-sel approach with k = 10 nearest-neighbors [36].

### 2.5 Multi-omics data integration

Co-inertia analysis (CIA) was performed using R package *omicade4* [37], to estimate the co-variability of proteomics and metabolomics datasets. Before CIA, each dataset was log2 transformed and Pareto scaled. The similarity between the two datasets was estimated with the RV parameter, which is a multivariate extension of the Pearson correlation coefficients. RV value close to 1 indicates a high degree of co-structure in datasets. The permutation test with 200 iterations was used to assess the significance of the RV coefficient.

### 2.6 Oil red O staining

Liver tissue samples of 3-day-old piglets were fixed in PBS-buffered 4% PFA for 48 h, immersed in sucrose (each 2 h in 7.5% and 15% sucrose at room temperature, followed by 30% sucrose over night at 4 °C), embedded in Tissue-Tek® O.C.T.™ compound, frozen on dry ice, and stored at −80 °C till cryosectioning. 4 μm thick cryosections were stained with oil red O stain and embedded in Kaiser's glycerin gelatin.

### 2.7 Clinical chemistry and determination of HOMA-IR and QUICKI index

For clinical-chemical analysis, frozen plasma samples derived from non-fasted 3-day-old piglets were thawed for 1 h at room temperature, mixed thoroughly and then centrifuged (10 min, 5000×*g* at 8 °C) and afterwards analyzed immediately using an AU480 clinical chemistry analyzer (Beckman Coulter) and adapted reagent kits from Beckman Coulter, Randox (Glycerol) or FUJIFILM Wako Chemicals Gmbh (NEFA) as described previously [38]. Insulin concentration was determined with ultrasensitive insulin ELISA from EDTA plasma (#10-1132-01, Mercodia) collected from newborn piglets before first milk intake. The homeostatic model assessment for insulin resistance index (HOMA-IR) [39] for estimating insulin resistance at fasting conditions was calculated using the formula: HOMA-IR = fasting plasma insulin (μU/mL) × fasting plasma glucose (mg/dL)/405. The ‘QUantitative Insulin sensitivity ChecK’ (QUICKI) index [40] was calculated with the formula: QUICKI = 1/[log(insulin (mU/L)) + log(glucose (mg/dL))].

### 2.8 Statistical analysis

All statistical analysis and data visualization were performed in R (https://www.r-project.org/). Statistical significance of proteome, metabolome, lipidome and clinical parameter changes was evaluated using two-way analysis of variance (ANOVA) considering the effect of the group (PHG/PNG), sex (female/male) and interaction between group and sex (group∗sex). All resulting p-values (group, sex and group∗sex) were pooled and adjusted for multiple-hypothesis testing with the Benjamini-Hochberg procedure. Biomolecules with a significant interaction effect were further followed by Tukey's honest significant difference (HSD) post-hoc test. Principal component analysis (PCA) was performed on log2 transformed data using prcomp() function in R. Hierarchical clustering was performed using the R package *ComplexHeatmap* [41] with Ward's method as the clustering method and the Euclidean as a distance measure. Supervised clustering method, orthogonal projection to latent structures discriminant analysis (OPLS-DA), according to the class information (PHG versus PNG), was performed using the R package *ropls* [42]. Before the OPLS-DA, omics datasets were log2 transformed and subsequently Pareto scaled (mean-centered and divided by the square root of standard deviation). The leave-one-out cross-validation (LOOCV) of all models was used to select the best fitted OPLS-DA model. LOOCV is advantageous for small datasets as it maximizes the size of the training set. R2Y and Q2Y were used to assess the fitting validity and predictive performance of the OPLS-DA model, respectively. A 200-step permutation test was employed to estimate whether the supervised classification according to the known class (PHG versus PNG) is significantly better than any other random classification. Variance importance in projection (VIP) scores of the selected OPLS-DA model were used to rank the metabolites based on their discriminating ability of PHG and the PNG. Over-representation analysis (ORA) based on significantly changed proteins was performed using the R package *webgestaltR* [43] with the functional category “GO Biological Process nonredundant”. The false-discovery rate was controlled using the Benjamini-Hochberg method. Western blot signal intensities, the homeostatic model assessment-insulin resistance index (HOMA-IR) and the ‘QUantitative Insulin sensitivity ChecK’ (QUICKI) index were compared using two-tailed Student's *t-*test.

## 3 Results

### 3.1 General aspects

**Figure 1:**
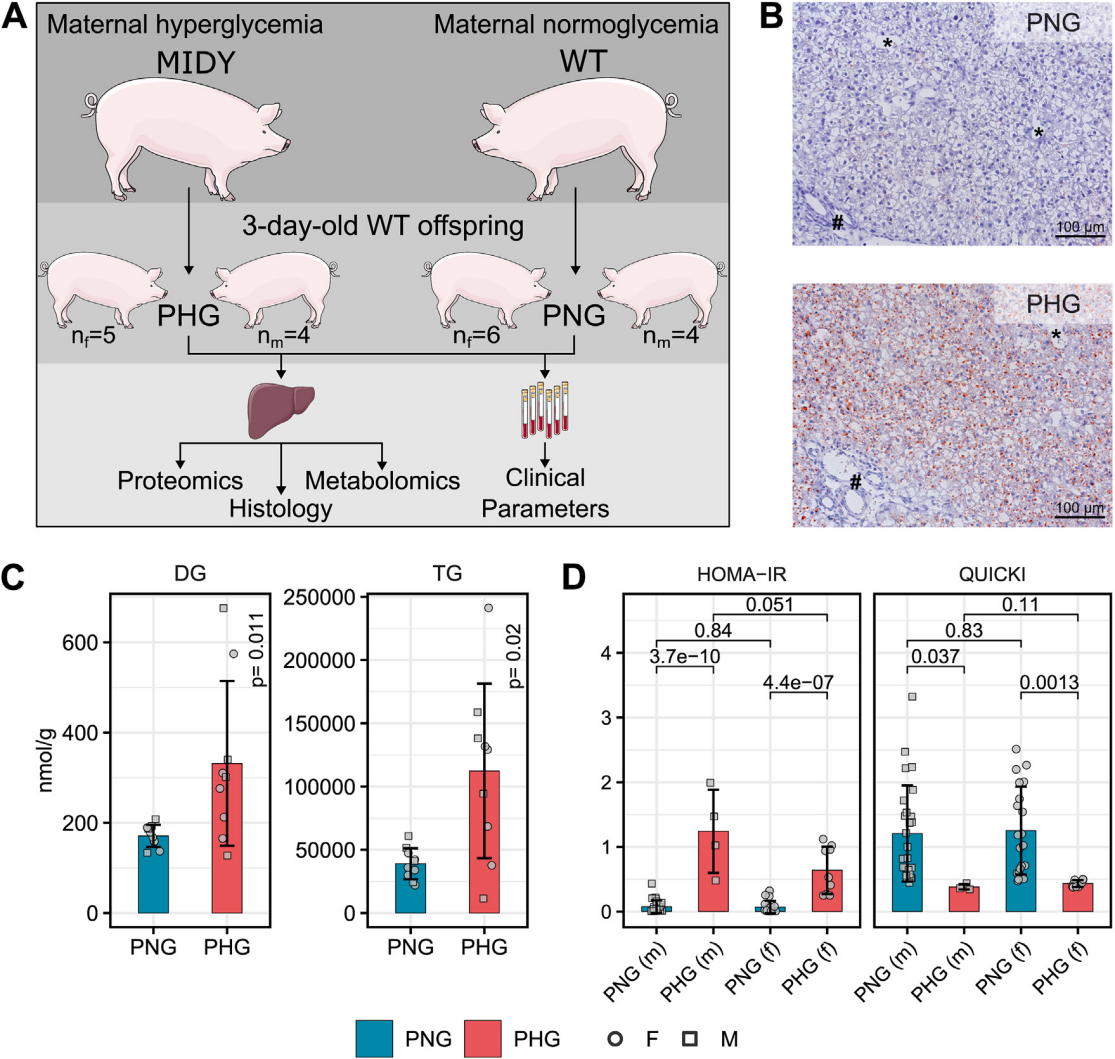
Experimental design, Oil red O stains of liver and assessment of insulin sensitivity. **A:** Proteomics, metabolomics and histological evaluation of liver samples alongside serum clinical chemical parameters from PHG (n = 5 female, n = 4 male) and PNG (n = 6 female, n = 4 male). PHG, piglets born to hyperglycemic mothers; PNG, piglets born to normoglycemic mothers; MIDY, mutant *INS* gene induced diabetes of youth; WT, wild-type. **B:** Oil Red O stains of liver cryosections of 3-day-old piglets show mediovesicular lipid accumulation in hepatocytes in PHG. #, portal triad; ∗, central vein. **C:** Total diglyceride (DG) and triglyceride (TG) levels in PHG and PNG. P-values are from two-way ANOVA (group effect). **D:** Homeostatic model assessment of insulin resistance (HOMA-IR) and the ‘QUantitative Insulin sensitivity ChecK’ (QUICKI) index of PHG (n = 9 female, n = 4 male) and PNG (n = 20 female, n = 26 male) at birth. Statistical significance of the pair-wise differences was assessed using the Student's *t*-test. Bar diagrams show means and standard deviations.

This study aimed to investigate the effect of maternal diabetes on non-diabetic offspring. For this purpose, as a translational model for human research, we used a non-obese genetically diabetic (*INS*^C94Y^ transgenic) pig model characterized by severe hyperglycemia [19], mimicking severe insulin deficient diabetes (SIDD) [44]. In this study, the hepatic proteome, metabolome as well as serum clinical parameters from 3-day-old wild-type (WT) piglets born to hyperglycemic mothers (PHG) were compared to the profiles of WT controls born to normoglycemic mothers (PNG). To complement the molecular findings, a histomorphological evaluation of the liver was performed (Figure 1A). Oil red O staining showed that PHG livers contained an increased amount of microvesicular and mediovesicular lipid droplets in hepatocytes (Figure 1B). To gain further molecular insights into an elevated lipid droplet formation, hepatic triglyceride (TG) and diglyceride (DG) levels were quantified using targeted lipidomics. Results showed elevation of both TG and DG levels in PHG liver (Figure 1C, Supplementary Fig. 2B). A detailed overview of the lipidomics results can be found in Supplementary Tables 1A–G and Supplementary Figs. 2A–C. Furthermore, homeostatic model assessment of insulin resistance (HOMA-IR) index monitored shortly after birth was higher in PHG (mean [SD], male: 1.24 [0.65], female: 0.64 [0.37]) than in PNG (mean [SD], male: 0.08 [0.09], female: 0.07 [0.09]). Consistently, quantitative insulin sensitivity check index (QUICKI) was lower in PHG (mean [SD], male: 0.38 [0.04], female: 0.43 [0.05]) compared with PNG (mean [SD], male: 1.20 [0.74], female: 1.25 [0.68]). QUICKI in PHG was below the cut-off value of 0.45 indicative for decreased insulin sensitivity (Figure 1D). The body weight of PHG was significantly lower than PNG (Supplementary Fig. 3A). Liver mass, relative to body weight, was not significantly different between the groups (Supplementary Fig. 3B). Neither sex nor group∗sex interaction-related differences were observed for these parameters.

### 3.2 Overview of proteome findings in the liver

**Figure 2:**
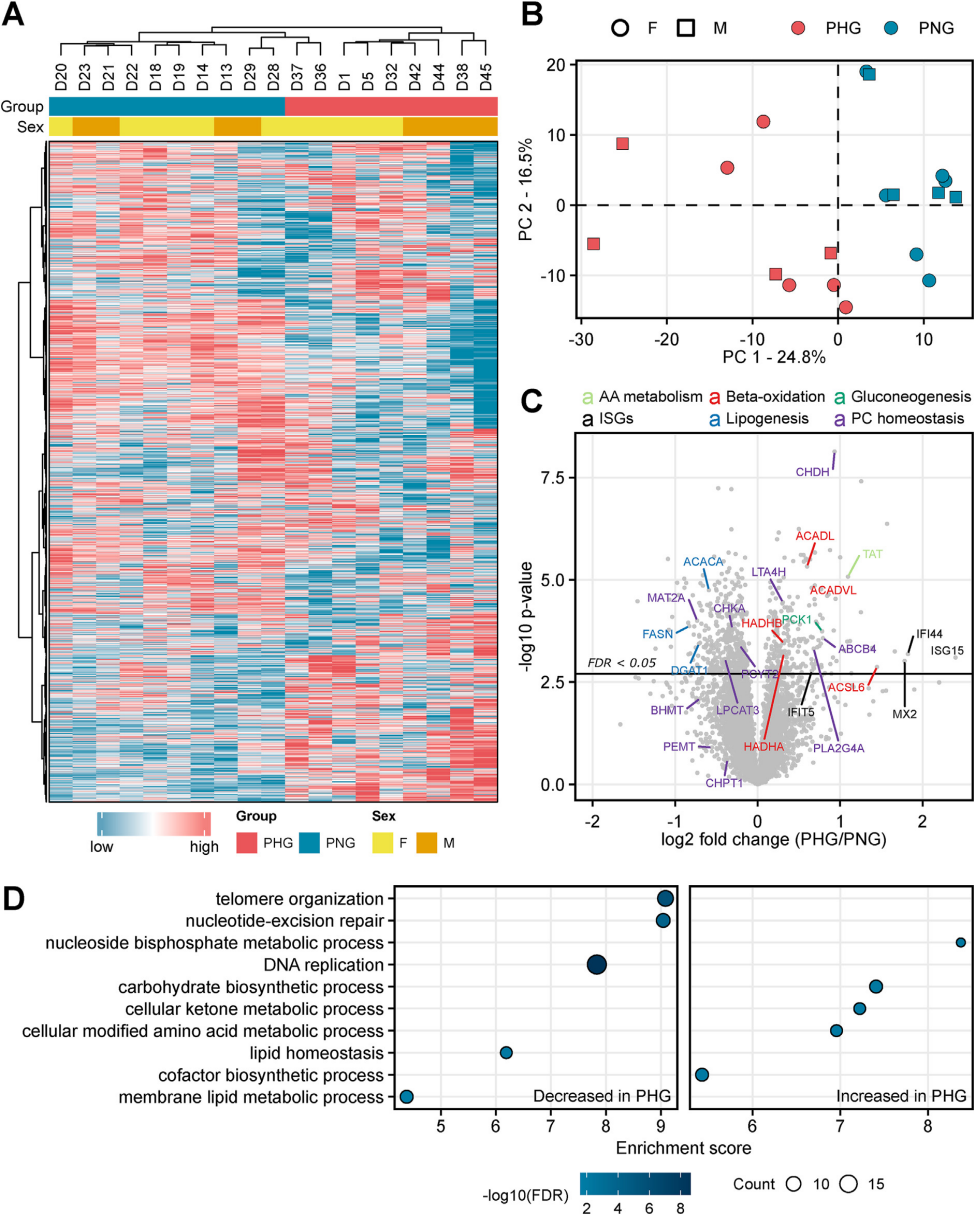
Overview of proteome differences in the liver from hyperglycemia exposed and control offspring. **A:** Unsupervised hierarchical clustering of standardized LFQ intensity values of liver proteomes leads to clustering of each sample according to the maternal glycemic status. The color code shows standardized abundance values. **B:** Principal component analysis of log2-transformed data reveals maternal glycemic status as the strongest contributor to the inter-sample variation of the liver proteomes. The shape of each spot corresponds to the sex, and the color to the mother's genotype. **C:** Volcano plot comparing the protein abundance change between conditions (PHG/PNG). The x and y axis show the log2 fold-change in protein levels and the log10 two-way ANOVA group p-value, respectively. Selected proteins are annotated with gene names and color coded according to the corresponding biological processes. Proteins changed in abundance with false-discovery rate of 0.05 are above the vertical solid line. ISGs, interferon stimulated genes; FDR, false discovery-rate **D:** Enrichment analysis results of liver proteins less abundant in PHG (left column) and more abundant in PHG liver (right column). The size of each dot indicates the number of differentially abundant proteins involved in the corresponding GO biological process (referred to as count in the figure) and colors the significance (Benjamini-Hochberg adjusted p-value) of enrichment. Enrichment score is the magnitude of over-representation.

To detect effects of maternal hyperglycemia on offspring's liver proteome, we performed a label-free liquid chromatography-tandem mass spectrometry analysis (LC-MS/MS) of liver tissue samples from PHG and PNG. To facilitate accurate and in-depth quantitative proteomics, a data-independent acquisition (DIA) approach was chosen. In the workflow, peptides were identified using an *in silico* predicted library, which was further refined by the project-specific chromatogram libraries generated with narrow-isolation window gasphase fractionation (GPF) DIA runs. The dataset has been submitted to the ProteomeXchange Consortium via the PRIDE [45] partner repository with the dataset identifier PXD040305. A total of 61,283 unique peptides from 6,313 protein groups were identified with high confidence (false-discovery-rate <0.01). Supplementary Table 2A contains a full list of all identified proteins and their abundance levels. In the unsupervised hierarchical clustering (Figure 2A) and principal component analysis (Figure 2B), the proteome profiles of liver tissue from PHG differed substantially from those of PNG, suggesting group-specific alterations in protein abundance.

To identify differentially abundant proteins, a two-way ANOVA was performed (Supplementary Table 2B). 123 proteins were found to be differentially abundant (Benjamini-Hochberg adjusted p-value ≤0.05 and l2fc ≥ 1.5) by the effect group (PHG/PNG), of which 62 were increased and 61 decreased in abundance (Supplementary Table 2C, Figure 2C). The protein with the highest increase in abundance in the PHG liver was ISG15 ubiquitin like modifier (ISG15) (5.3-fold). Likewise, the levels of other proteins involved in interferon signaling pathway such as interferon-induced GTP-binding protein Mx2 (MX2), interferon induced protein 44 (IFI44), and interferon induced protein with tetratricopeptide repeats 5 (IFIT5) were elevated in PHG samples. Moreover, proteins involved in glucose metabolism, such as phosphoenolpyruvate carboxykinase (PCK1) and glucose-6-phosphate isomerase (GPI) were increased in abundance. Several proteins involved in retinol metabolism, such as retinol-binding protein 4 (RBP4) and dehydrogenase/reductase 7B (DHRS7), were also elevated. Further proteins with increased abundance in PHG liver were tyrosine aminotransferase (TAT), branched-chain-amino-acid aminotransferase (BCAT1), and aromatic-l-amino-acid decarboxylase (DDC), all known to be involved in amino acid metabolism. A large fraction of up-regulated proteins is known to be involved in lipid homeostasis, among which are acyl-CoA synthetase long chain family member 6 (ACSL6), long-chain specific acyl-CoA dehydrogenase (ACADL), mitochondrial acyl-CoA dehydrogenase very long chain (ACADVL), propionyl-CoA carboxylase alpha and beta chain (PCCA and PCCB), and others. Furthermore, proteins involved in glycerophospholipid metabolism (e.g. choline dehydrogenase (CHDH) and phospholipase A2 (PLA2G4A)) and transport (e.g. ATP-binding cassette 4 (ABCB4)) were elevated in abundance. On the other hand, some of the down-regulated proteins are also known to be involved in lipid metabolism, among others fatty acid synthase (FASN), O-acyltransferase (DGAT1), acetyl-CoA carboxylase 1 (ACACA), ceramide synthase 4 (CERS4), and others. Furthermore, S-adenosylmethionine synthase (MAT2A), a protein involved in the methionine cycle, was decreased in abundance.

To get functional insights from proteome alterations between PHG and PNG, over-representation analysis (ORA) was performed using WebGestalt. The detailed results of the enrichment analysis are provided in Supplementary Table 2D and Figure 2D. Briefly, proteins involved in the nucleoside bisphosphate metabolic process, carbohydrate metabolic process, cellular ketone metabolic process, cellular modified amino acid metabolic process, and cofactor biosynthetic process were significantly overrepresented in the set of up-regulated proteins, while proteins involved in DNA replication, regulation of plasma lipoprotein particle levels, telomere organization, nucleotide-excision repair, DNA replication, lipid homeostasis, and membrane lipid metabolic process were overrepresented in the set of down-regulated proteins.

In terms of sex-related differences, only UDP-glucuronosyltransferase was changed significantly and was elevated in the liver of female compared to male offspring (Supplementary Table 2E). To explore proteins changed in the offspring's liver due to maternal glycemia in a sex-dependent manner, the group∗sex interaction effect from the two-way ANOVA was used. This revealed only a few proteins that were significantly influenced by the group∗sex interaction effect (Supplementary Fig. 4, Supplementary Table 2F). The proteins most significantly affected by the interaction effect were vacuolar protein sorting-associated protein 41 homolog (VPS41) and 60S ribosomal protein L26-like 1 isoform X1 (RPL26L1), both increased in female PHG (compared to female PNG) but decreased in male PHG (compared to male PNG). A similar regulation pattern was observed for further proteins like glutaredoxin 5 (GLRX5) and complement component 1 Q subcomponent-binding protein, mitochondrial (C1QBP).

Furthermore, to confirm quantitative changes detected by mass spectrometry by other means of quantification, we selected three candidates where working porcine-specific antibodies were available and quantified them using Western blot. Supplementary Fig. 5 shows the abundance change of formyltetrahydrofolate dehydrogenase (ALDH1L2), Ras-related protein Rab-3 (RAB3D) and claudin (CLDN15) between PHG and PNG and they are in line with our mass spectrometry-based quantitative data.

### 3.3 Protein–protein interaction construction

**Figure 3:**
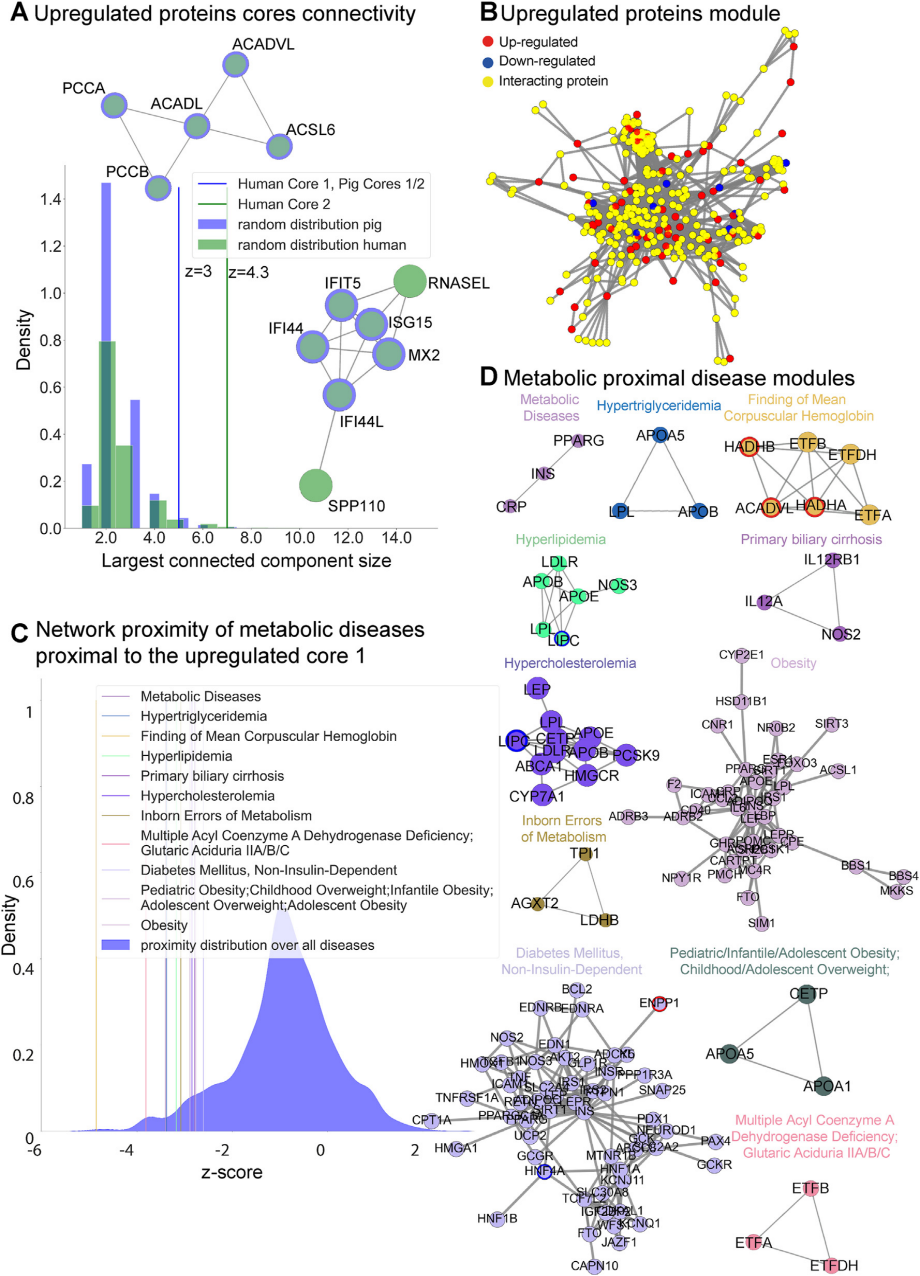
Network analysis of differentially abundant proteins and their relationships with human diseases. **A:** Identification of two up-regulated core proteins that deviate from random expectation (10,000 random sets of proteins of equal size) based on their connectivity (z-score_core1_ = 3, z-score_core2_ = 4.3). The green color refers to the human PPI, and the blue color to the pig PPI. **B:** Up-regulated expanded network which contains all up-regulated proteins and their interacting partners. This subnetwork is formed by 312 proteins colored in: red if up-regulated in PHG, blue if down-regulated in PHG, and yellow if not detected but interacting with differentially abundant proteins. **C:** Network proximity z-score distribution for all diseases (N = 11,099). With vertical, colored lines are highlighted 15 metabolic diseases that have been observed to be particularly proximal (related) to the up-regulated core 1. **D:** Diseases network modules (network size >2 nodes) for proximal metabolic disorders to up-regulated core 1. Each node color reflects each specific disease, and a red outline is marked if a protein was identified as overly abundant in PHG, or a blue outline if it was down-regulated in PHG.

Next, we evaluated whether among the differentially abundant proteins we could identify subsets of highly interacting proteins that participate in the same molecular mechanisms and tried to relate these mechanisms with human pathology. To do so, we first generated a pig-specific and a human-specific protein–protein interaction network (PPIs) compiled from the STRING database v11.5 (https://string-db.org/) [27], obtaining 15,360 nodes and 170,244 edges, and 16,793 proteins and 251,982 edges respectively. At this point, we compared the size of the connected components of the differentially abundant proteins against 10,000 random groups of proteins of equal size. In this way, we were able to identify two main cores among the up-regulated proteins in both pigs and humans (Figure 3A). The first module consists of five interacting proteins (ACADVL, ACADL, ACSL6, PCCB, PCCA) conserved in pig (p-value = 0.001) and in human (p-value = 0.003), which is responsible for lipid homeostasis (Supplementary Table 3A contains the full list of significantly enriched terms (adjusted p-value <0.05)). The second up-regulated core is formed of five proteins in pig (MX2, IFIT5, IFI44, IFI44L, ISG15) (p-value = 0.001) and seven in human (MX2, IFIT5, IFI44, IFI44L, ISG15, SP110, RNASEL) (p-value = 1e-05), which is related to an interferon type I response (full list of enriched terms Supplementary Table 3B for pig and 3C for human). In both species, the down-regulated proteins form a connected core, 17 proteins in pigs (p-value = 1.7e-60), and 22 in humans (p-value = 3e-74) (Supplementary Fig. 6A).

Based on the fact and our observation that pigs and humans share similar core mechanisms on a network level, we decided to focus on the latter. Using a random walk with restart algorithm (see methods), we identified a network of 312 up-regulated proteins and their interactors (Figure 3B) and another network for 363 proteins which were down-regulated in PHG (Supplementary Fig. 6B). These two networks are very different as shown by their poor edge overlap (Jaccard index = 0.002), proving once again that they lay in two different parts of the PPI network contributing to different biological mechanisms. Their enrichment analysis resembles our previous findings (Supplementary Tables 3D–E) pointing to those interacting proteins that in tandem with the differentially abundant ones contribute to specific phenotypes (i.e. “fatty acid degradation”). Finally, to check the relationship of these dysregulated proteins with disease onset, we extracted disease–gene associations from DisGeNet [31], leading to a list of 11,099 diseases (after filtering). We computed two measures: the Jaccard index between the set of perturbed genes in diseases and the dysregulated proteins in our set-up and the network proximity [32] (Supplementary Table 3F). Since the Jaccard index does not make use of any network properties, these relationships can be driven even by a very small pool of genes. To address this, we decided to pursue our analyses by using network proximity and identified a plethora of related diseases (227, for the first up-regulated core, and 1,275, for the second one) (adjusted p-value <0.05, Supplementary Table 3G). Among the 227 proximal diseases to the first up-regulated core, we focused on those relevant to metabolic disorders and liver diseases (Figure 3C), observing very small z-scores compared to those of all diseases, standing for their closeness to the up-regulated core in the human PPI. The genes known to be responsible for these pathological conditions are strictly related to lipid metabolism, such as lipoprotein lipase (LPL), its receptor (LPLR), and hepatic triacylglycerol lipase (LIPC) (Figure 3D). This tight distance in the human PPI suggests that frequently reported susceptibility of GDM offspring to childhood and adolescence overweight may be caused by the network pathways that connect the up-regulated core genes (PCCA, PCCB, ACADL, ACADVL, ACSL6), to APOA5, CETP, and APOA1 (Figure 3D). Similar considerations can be applied to the second up-regulated core (related to the IFN pathway) and to the expanded unified up-regulated core (Supplementary Table 3G). After expansion, also by using the Jaccard index measure, we could observe among the most statistically significant associated diseases, primary and secondary biliary cholangitis (Benjamini-Hochberg adjusted p-value: 0.03), Glutaric Aciduria II (type A, B, C) (Benjamini-Hochberg adjusted p-value: 0.004), and Multiple Acyl Coenzyme A Dehydrogenase Deficiency (Benjamini-Hochberg adjusted p-value: 0.004), which could hint changes in bilirubin metabolism due to perturbations of the immediate neighbors in the human PPI of the up-regulated proteins.

### 3.4 Overview of metabolome findings in the liver

**Figure 4:**
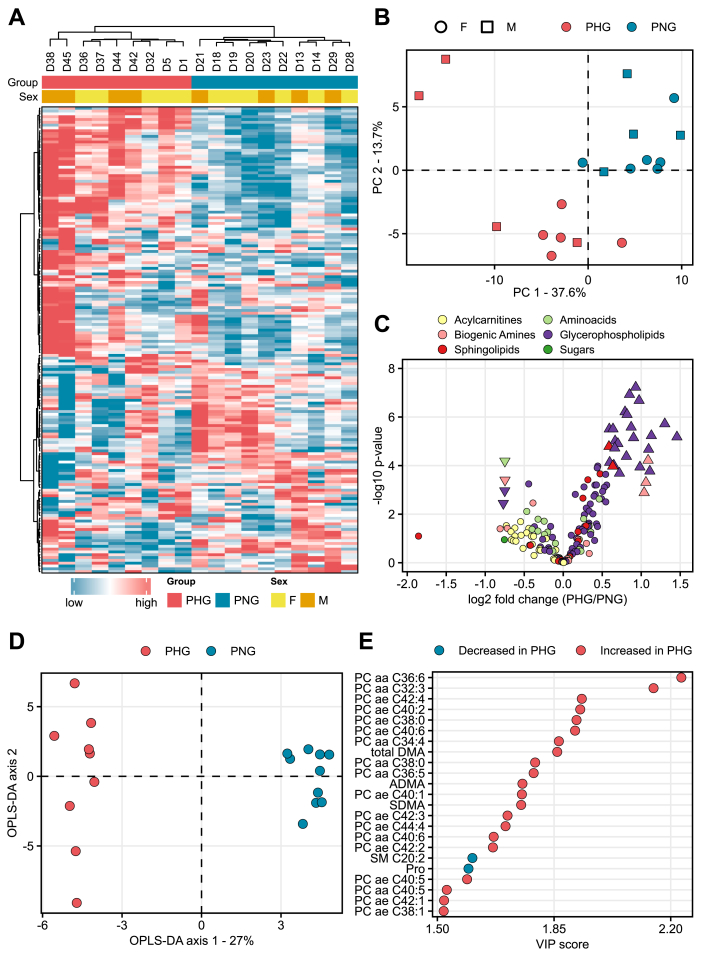
Overview of metabolome differences in the liver from hyperglycemia exposed and control offspring. **A:** Unsupervised hierarchical clustering of metabolite levels (pmol/mg tissue) leads to the clustering of each sample according to the maternal glycemic status. The color code shows standardized abundance values. **B:** Principal component analysis of log2 transformed and unit variance scaled data reveals maternal glycemic status as the strongest contributor to the inter-sample variation of the liver metabolome. The shape of each spot corresponds to the sex, and the color to the mother's genotype. **C:** Volcano plot comparing the metabolite abundance change between conditions (PHG/PNG). Significantly changed metabolites (Benjamini-Hochberg adjusted p-value ≤0.05 and fold change ≥1.5) are shown as up- and down-pointing triangles for increased and decreased abundance in PHG versus PNG, respectively. Circles correspond to non-significant changes. The x and y axis show the log2 fold-change in metabolite levels and the log10 two-way ANOVA group p-value, respectively. Different metabolite classes are color-coded. **D:** Supervised classification of PHG from PNG samples using the cross-validated orthogonal partial least squares discriminant analysis (OPLS-DA). The x and y axis show the predictive (between class separation) and orthogonal component (within class separation), respectively. The best fitted OPLS-DA model was selected based on leave-one-out cross-validation followed by permutation test with a 200-step iteration which yielded R2X = 0.58, R2Y = 0.99, Q2 = 0.93 (R2Y p = 0.002, Q2, p = 0.002). **E:** Variance importance on projection (VIP) plot. Metabolites with the strongest impact on the supervised classification of PHG and PNG samples were extracted from the OPLS-DA model. Metabolites with VIP >1.5 are shown.

**Figure 5:**
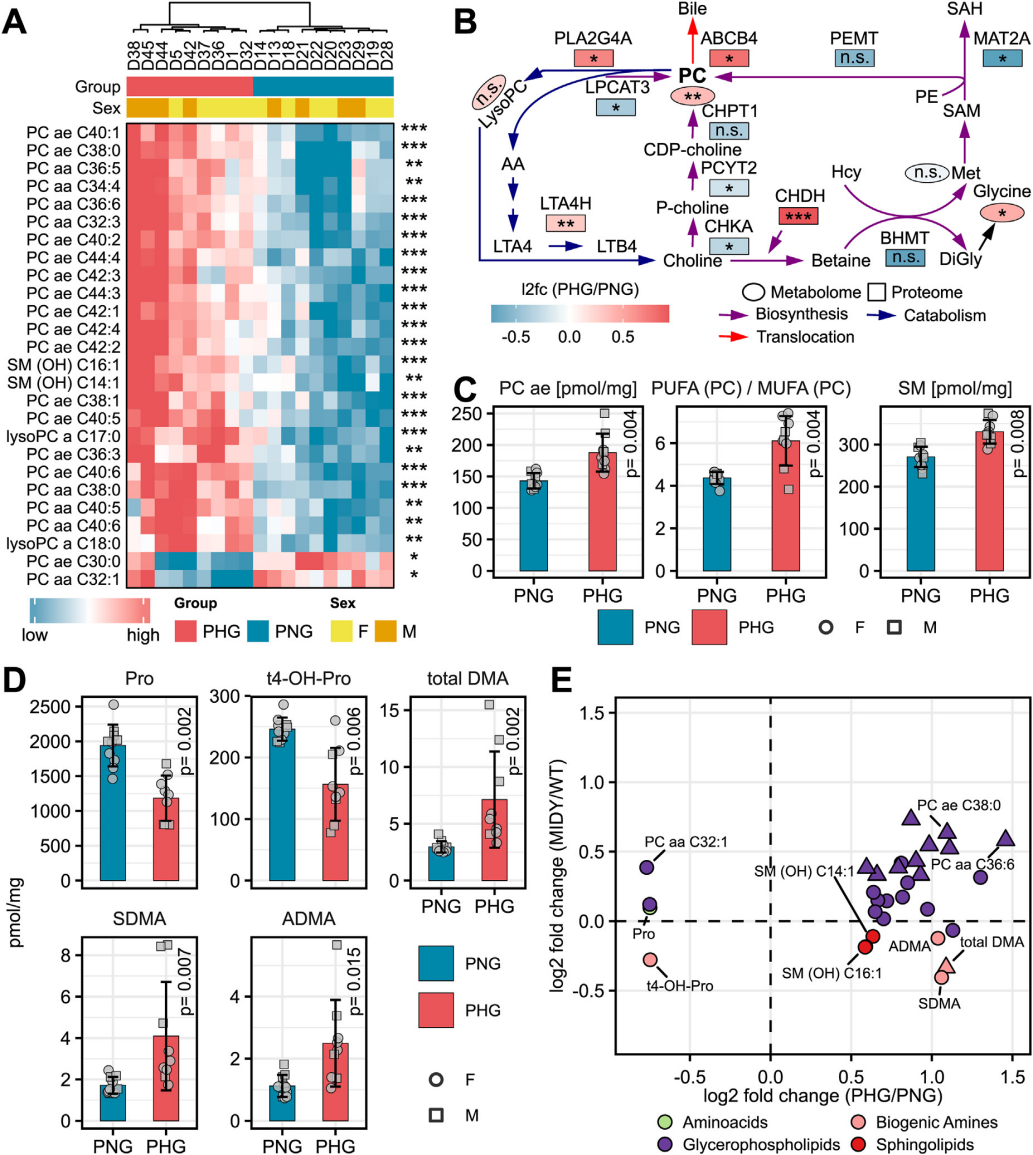
Overview of significantly changed metabolites between PHG and PNG. **A:** Levels of the significantly changed glycerophospholipids and sphingolipids in PHG and PNG samples. **B:** Scheme of PC synthesis (violet arrows), breakdown (dark blue arrows) and removal (translocation to bile, red arrow). Metabolites and enzymes are depicted as ellipses and rectangles, respectively. LysoPC, lysophosphatidylcholine; PLA2G4A, phospholipase A2 group IVA; LPCAT3, lysophosphatidylcholine acyltransferase 3; SM, sphingolipid; AA, arachidonic acid; LTA4, leukotriene A4; LTA4H, leukotriene A4 hydrolase; LTB4, leukotriene B4; CHKA, choline kinase alpha; PCYT2, phosphate cytidylyltransferase 2, ethanolamine; CDP, cytidine 5′-diphosphocholine; PC, phosphatidylcholine; CHDH, choline dehydrogenase; CHPT1, choline phosphotransferase 1; ABCB4, ATP-binding cassette 4; BHMT, betaine-homocysteine S-methyltransferase; DiGly, diglycine; Hcy, homocysteine; Met, methionine; SAM, S-adenosyl methionine; SAH, S-adenosylhomocysteine; PEMT, phosphatidylethanolamine n-methyltransferase; MAT2A, methionine adenosyltransferase 2A; PE, phosphatidylethanolamine; n.s., not significant. **C:** Levels of the significantly changed (Benjamini-Hochberg adjusted p-value ≤0.05) metabolite ratios. PUFA, polyunsaturated fatty acid; MUFA, monounsaturated fatty acid. **D:** Bar diagram of amino acids and biogenic amines significantly changed between groups. The bar diagrams show means and standard deviations. P-values are from two-way ANOVA (group effect) after the Benjamini-Hochberg correction. **E:** Scatter plot of log2 fold-change of significantly altered metabolites (PHG/PNG) in offspring liver versus the changes of the same metabolites in the insulin-deficient diabetes (MIDY) versus WT pig plasma [46]. The mother of the offspring used in this study has the same insulin mutation as MIDY pigs published previously [19]. Different metabolite classes are color-coded. Metabolites which were changed in abundance between MIDY versus WT with a p-value <0.05 are shown as triangles. Benjamini-Hochberg adjusted significant p-values are labelled with asterisks in A and B, ∗p < 0.05; ∗∗p < 0.01; ∗∗∗p < 0.001.

To gain further insights into the alteration of metabolic pathways as revealed by proteomics, quantitative readouts of relevant metabolite classes were performed. The results of the targeted metabolomics analysis are shown in Supplementary Table 4A. Hierarchical clustering (Figure 4A) and principal component analysis (Figure 4B) separated samples of PHG and PNG. To reveal metabolites changed by the effects of group, sex, and group∗sex, a two-way ANOVA was performed (Supplementary Table 4B). Metabolites with Benjamini-Hochberg adjusted p-value ≤0.05 and l2fc ≥ 1.5 were considered significant. 31 metabolites were changed by the effect group (Supplementary Table 4C, Figure 4C). The supervised OPLS-DA method was used to evaluate to what extent metabolomics data can discriminate PHG from PNG. OPLS-DA clearly separated groups (Figure 4D). Statistical evaluation of the OPLS-DA indicated a robust model (R2X = 0.58, R2Y = 0.99, Q2 = 0.93). The permutation test with 200 iterations showed the significance of both predictive (Q2Y) and fitting (R2Y) components (p = 0.002). Variable importance in projection (VIP) plot (Figure 4E) revealed metabolites with the highest contribution to the separation of PHG from PNG animals on the OPLS-DA plot. Figure 5 provides a detailed overview of differentially abundant metabolites. 24 different glycerophospholipids (specifically phosphatidylcholines (PC)) were changed in abundance of which 22 were increased and only two (PC ae C30:0 and PC aa C32:1) were decreased (Figure 5A). Furthermore, two sphingolipids (SM (OH) C14:1 and SM (OH) C16:1) were elevated (Figure 5A). In the PHG liver, enzymes and metabolites involved in the breakdown and removal (translocation from hepatocytes to bile) of the PC were elevated while those involved in biosynthesis were reduced (Figure 5B–C). Several members of biogenic amines were changed in abundance between PHG and PNG, of which total DMA (dimethylamine), SDMA (symmetric dimethylarginine) and ADMA (asymmetric dimethylarginine) were elevated while trans-4-hydroxyproline (t4-OH-Pro) was reduced. Furthermore, the amino acid proline was reduced by 1.7-fold (Figure 5D). Only one metabolite, PC ae C42:4, was affected by the effect sex (decreased in female offspring) (Supplementary Table 4D) and only two metabolites (PC ae C42:3 and SM C26:0) were affected by the interaction group∗sex (Supplementary Table 4E, Supplementary Fig. 7). Only three metabolite ratios, poly-unsaturated to mono-unsaturated glycerophosphocholines (PUFA (PC)/MUFA (PC)), total PC ae and total sphingolipid (SM), were significantly changed between PHG and PNG (Benjamini-Hochberg adjusted p-value ≤0.05) and were elevated in PHG liver (Supplementary Tables 4F–G, Figure 5C). To check for similarities between the metabolic alterations in offspring and mother, plasma metabolomics data from this study was compared to a previously published set of plasma metabolite alterations from MIDY versus WT pigs [46] (Figure 5E). The mother of the offspring used in this study had the same insulin mutation as MIDY pigs published previously [19]. The majority of PCs changed in abundance in the offspring were also significantly changed in the same direction in the MIDY versus WT plasma. Total DMA was elevated in the offspring liver while it was significantly reduced in the MIDY plasma. Proline which was significantly increased in the offspring liver was not significantly changed in the MIDY plasma. The same is true for sphingolipids (SM (OH) C14:1 and SM (OH) C16:1) and biogenic amines (SDMA and ADMA, t4-OH-Pro).

### 3.5 Cross-omics correlation

**Figure 6:**
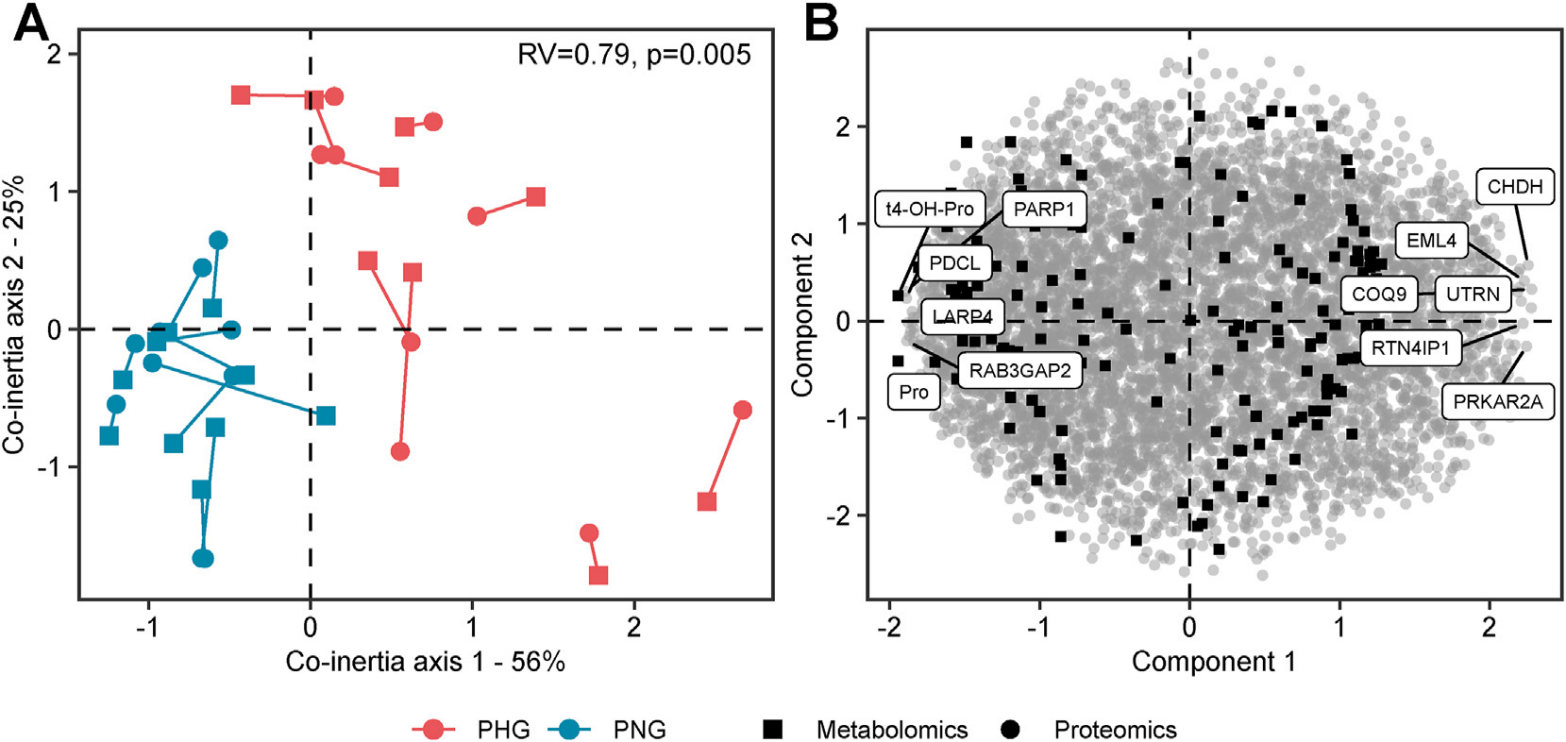
Proteomics and metabolomics data integration. (A, B) Multiple co-inertia analysis of proteome (circle) and metabolome (square) data from the PHG and PNG liver samples showing the first two components in the sample (A) and variable (B) space. The line length in the sample space (A) reflects the strength of the cross-omics correlation for each sample. The RV coefficient (RV = 0.79, 200 permutations, p = 0.005) shows the correlation between the two datasets. An RV close to 1 indicates a strong correlation. Proteins and metabolites with the highest values in component 1 are labelled in a variable space (B).

Using a co-inertia analysis (CIA) [47], we investigated the complex association between proteomics and metabolomics datasets. CIA projects multiple omics datasets simultaneously onto the same plane. Representation of samples on a lower-dimensional space reveals global co-variability between proteomics and metabolomics datasets (Figure 6A). CIA reveals that proteomics and metabolomics datasets are more similar within groups than between groups. The first component of the CIA (horizontal) accounted for 56% of the variance, and the second component (vertical) accounted for 25%. The CIA showed clear clustering of PHG and PNG samples. In line with this RV coefficient which represents the degree of association was 0.79 and was significant as revealed by 200-step permutation-based test (p = 0.005). The corresponding score plot shows the proteins and metabolites responsible for partitioning PHG and PNG samples on the CIA plot (Figure 6B). In the score plot, each quantified protein and metabolite is depicted by the black square and grey circles, respectively and some of the most informative biomolecules across datasets are labelled.

### 3.6 Overview of clinical-chemical findings in the serum

**Figure 7:**
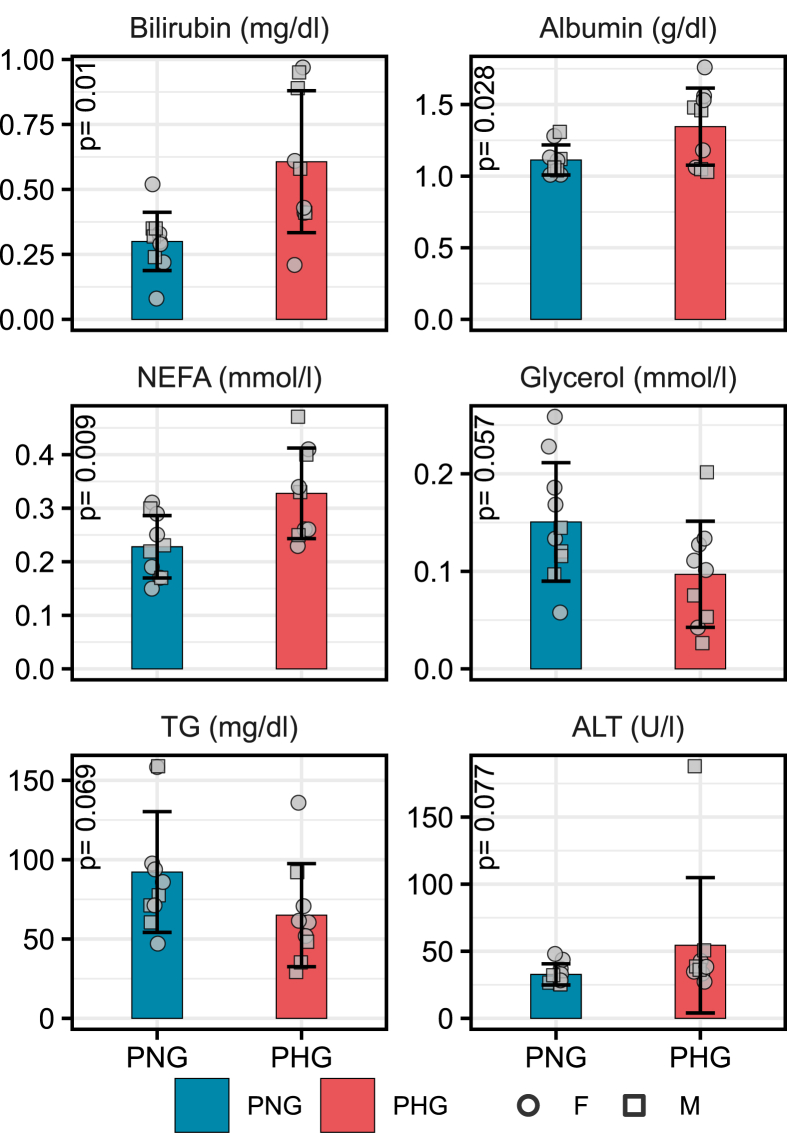
Changes in selected clinical-chemical parameters in the PNG and PHG blood. Bar diagrams show the mean and standard deviation. P-values are from two-way ANOVA (group effect). NEFA, non-esterified fatty acid; TG, triglyceride; ALT, alanine transaminase.

To clarify if maternal diabetes is associated with alteration of circulating biomarkers of liver damage, relevant clinical-chemical parameters were measured in the serum of PHG and PNG. The detailed clinical-chemical data and the results of the two-way ANOVA analysis are shown in Supplementary Table 5A and Supplementary Table 5B, respectively. Clinical-chemical parameters with statistically significant (p-value ≤0.05) changes between PHG and PNG serum samples were bilirubin (increased in PHG), non-esterified free fatty acids (NEFA) (increased in PHG), and albumin (increased in PHG) (Supplementary Table 5C). Glycerol (decreased in PHG, p = 0.06) and triglycerides (decreased in PHG, p = 0.07) levels were changed as a trend (Figure 7). High-density lipoprotein levels were significant for the effect of sex (increased in female offspring) (Supplementary Table 5D). Alanine transaminase (ALT) showed a significant interaction effect, with significantly higher levels in male PHG versus male PNG (Supplementary Table 5E, Supplementary Fig. 8).

## 4 Discussion

To investigate to what extent maternal hyperglycemia affects the offspring's liver metabolism, a multi-omics analysis combining data-independent acquisition proteomics and targeted metabolomics was performed. Additionally, relevant clinical-chemical parameters that reflect the liver state were measured in the serum. In this work, the liver and serum samples were collected from offspring born to a genetically engineered diabetic pig model for mutant *INS* gene-induced diabetes of youth (MIDY) [19] (PHG) and from offspring born to WT littermate controls (PNG), according to the principles of systematic random sampling [6]. The body weight of PHG was significantly lower than PNG. Similarly, in human studies, neonates of mothers with severe diabetic complications tended to have a lower birthweight (SGA) [48,49]. Like macrosomia, SGA is a risk factor for a variety of diseases in future life (reviewed in [50]). To clarify if hepatic damage in the offspring due to maternal glycemia is apparent already in the neonatal period, we investigated livers from 3-day-old piglets. To our knowledge, this is the first holistic multi-omics study from a clinically relevant large animal model addressing the molecular derangements in the offspring liver caused by maternal hyperglycemia.

Circulating bilirubin was significantly elevated in the offspring born to hyperglycemic mothers which was also observed previously in human offspring studies [51,52], underlining the clinical relevance of our finding. A higher level of bilirubin may reflect different types of liver or bile duct complications [53]. In line, disease–gene association revealed several diseases associated with disturbed bilirubin metabolism. One of the primary constituents of bile are phospholipids (predominantly phosphatidylcholines (PC)) [54]. PC excretion into bile is mediated by the PC-specific floppase ABCB4 [53,55]. Our targeted metabolomics revealed consistent elevation of multiple PC (with mainly one acyl- and one alkyl-bound fatty acids (PC ae), and a higher proportion of polyunsaturated PCs), while proteomics showed significantly elevated levels of ABCB4, suggesting an active translocation of PCs to bile. Translocation of PCs is considered to have hepatoprotective properties as PCs inactivate the detergent activity of bile salts to prevent damage to cell membranes [56]. Besides translocation to bile, hydrolysis of PCs by phospholipase A2 to produce fatty acids and a lysoPC is an important step in PC homeostasis [57]. The products of PC hydrolysis are important precursors for generating key inflammatory mediators, oxylipins [58]. In our data, phospholipase A2 was significantly elevated while one of the downstream enzymes leukotriene a4 hydrolase (LTA4H) was moderately increased (LTA4H, l2fc = 0.31, adjusted p-value = 0.006), suggesting a breakdown of PC molecules and generation of leukotrienes in PHG liver. Finally, PC homeostasis in the liver is achieved via the metabolic pathways involved in its biosynthesis, predominantly from choline via the CDP-choline pathway (also known as the Kennedy pathway) [59]. Choline kinase (CHKA), the initial enzyme in the sequence, catalyzes the transfer of a phosphate group from adenosine triphosphate (ATP) to choline to form phosphocholine. Subsequently, the key regulatory enzyme in this process, CTP:phosphocholine cytidylyltransferase (PCYT2, alias CCT) catalyzes the transfer of a cytidylyl group to phosphocholine to form CDP-choline, which then forms PC (catalyzed by choline phosphotransferase 1 (CHPT1 alias CPT1)). Although with a moderate fold change (CHKA, l2fc = −0.31; PCYT2, l2fc = −0.22), CHKA and PCYT2 were reduced significantly (adjusted p-value <0.05). CHPT1 levels were also reduced but did not reach statistical significance. CDP-choline is the major pathway of PC synthesis, however, in the hepatocytes where PC demand is high, it can also be synthesized by sequential methylations of phosphatidylethanolamine (PE) where MAT2A-catalyzed S-adenosyl-methionine (SAM) transformation to S-adenosylhomocysteine (SAH) donates the methyl groups. We found significantly reduced levels of MAT2A together with non-significantly reduced levels of other enzymes involved in this pathway. Additionally, LPCAT3, which catalyzes the third mechanism of PC synthesis - reacylation of lysoPC to PC [60] - was moderately reduced (l2fc = −0.4, adjusted p-value = 0.03). Collectively, our data show reduced levels of enzymes involved in PC synthesis, but elevated levels of enzymes and downstream products involved in its elimination and breakdown. Elevated PC levels in the PHG, despite reduced biosynthesis, may be explained by increased transplacental transfer from the hyperglycemic mother and subsequent hepatic uptake. This is in line with the previous data where PC levels were shown to be elevated in the serum of hyperglycemic pigs [46]. We suggest that the feedback loop mechanism by which increased PC levels downregulate enzymes involved in its biosynthesis is plausible. Supporting our hypothesis, previous reports showed a correlation of maternal and fetal metabolites during both the peripartum period [61] and even several years postpartum [62].

In line with increased lipid species as revealed by targeted metabolomics and targeted lipidomics, higher total hepatic lipid content was detected using Oil red O staining. Accumulation of liver fat is recognized as a risk factor for non-alcoholic fatty liver disease (NAFLD) [63], cardiometabolic disease [64,65] and other complications. Although the presence of liver steatosis in the offspring born to a diabetic mother is supported by several recent human [13,17,66,67] and rodent studies [68], another human study found that in predicting infant hepatic fat content, maternal diabetes may be less important than the presence of maternal obesity [14]. Authors of two systematic reviews proposed that the evidence for an association between maternal diabetes and offspring adiposity, which is strongly associated with NAFLD, remains inconclusive due to the attenuation of the association when adjusting for maternal pre-pregnancy BMI [69,70]. Lipogenesis as well as availability of plasma fatty acids are considered as important contributors to hepatic steatosis [71]. The initial rate-limiting step of hepatic *de novo* lipogenesis (DNL) is acetyl-CoA carboxylation to malonyl-CoA by the action of acetyl-CoA carboxylase (ACACA) [72]. Subsequent conversion of malonyl-CoA into palmitic or various other fatty acids is catalyzed by fatty acid synthase (FASN) which plays a central role in hepatic DNL [73]. The terminal step of triglyceride (TG) synthesis - the acylation of diglyceride - is catalyzed by diacylglycerol O-acyltransferase 1 (DGAT1) [74]. Despite increased hepatic fat content, levels of ACACA, FASN and DGAT1 were significantly reduced. Decreased circulating TG levels in PHG may be explained by an elevated hepatic TG accumulation and reduced release in the serum. This is in line with the downregulation of DGAT1 in PHG, as DGAT1 overexpression is associated with higher rates of very-low-density lipoprotein-TG complex secretion from rat hepatoma cells [75]. Conversely, inhibition of DGAT1 in mouse liver and isolated hepatocytes resulted in an increased transfer of fatty acids into mitochondria for beta-oxidation [[76], [77], [78]]. In PHG beta-oxidation markers such as ACSL6, ACADL and ACADVL were elevated suggesting active degradation of long-chain and very long-chain fatty acids. Furthermore, although with a moderate fold-change (l2fc = 0.32) both HADHA and HADHB which catalyze the last three steps of beta-oxidation were increased in abundance (adjusted p-value <0.05). Decreased lipogenic enzymes in the liver suggests that elevated hepatic lipid content in PHG is not linked to DNL. This is in line with the observation that limited capacity for DNL exists in human fetus, and the drivers of fetal fat accumulation are primarily supplied transplacentally [79]. Shifting the balance of lipid metabolism away from *de novo* synthesis to favor lipid breakdown via beta-oxidation, mechanistically resembles the observation made for PC (see above). Decreased DNL together with increased beta-oxidation might be a way of adaptation developed in offspring to slow down or prevent the progression of increased fat content into liver steatosis, which is especially relevant in pigs as they seem to be protected against steatosis even in morbid obesity [80]. In line, previous studies reported protection against steatosis through pharmaceutical inhibition of DNL enzymes [81,82]. The resistance of pigs to hypertriglyceridemia is not well understood but extrahepatic lipogenesis has been proposed as a potential mechanism [83]. Another key driver of reduced lipogenesis might be PC which were elevated in PHG (see above). An elevated lipogenesis and steatosis in early stages of fatty liver disease was shown in the setting of reduced PC [84]. Additionally, several clinical studies observed the attenuation of steatosis after treatment with PC (reviewed in [85]).

Besides lipid metabolism, the homeostasis of other key biomolecules such as amino acids and glucose is a pivotal function of the liver. Under normal circumstances, the fetus is dependent on a continuous supply of glucose from the mother, and no significant production of glucose (gluconeogenesis) by the fetus has been demonstrated [86]. Conversely, a rapid rise of hepatic gluconeogenesis is observed in newborn mammals in parallel with the appearance of PCK1, the key enzyme of this pathway [87]. Specifically, in humans, gluconeogenesis is apparent soon after birth in healthy newborns and it contributes 30% of the total glucose produced [88]. Our proteomics data revealed significantly higher levels of PCK1 in PHG liver. Increased levels of gluconeogenic precursors were observed in the plasma of piglets born to diabetic mothers [18], and was explained by reduced insulin sensitivity. Impaired insulin sensitivity was also observed in offspring exposed to hyperglycemia *in utero* due to maternal GDM or type 1 diabetes compared with offspring from the background population [89]. It was proposed that increased rates of gluconeogenesis in the offspring born to diabetic mothers may be predictive of the increased risk of glucose intolerance in later life [89]. Interestingly, PCK1 was elevated in the liver of the male but not in female offspring born to streptozotocin (STZ)-induced diabetic mice [90]. Similarly, in our study increase in PCK1 levels were almost three times higher in male than in female PHG when compared to PNG. We also observed significantly elevated circulating levels of ALT in the PHG male but not in female offspring. ALT catalyzes conversion of the main gluconeogenic precursor alanine into pyruvate for glucose production and thus plays an important role in gluconeogenesis [91]. An ALT blood test is used to diagnose liver disorders [92] and it has been shown that ALT activities are increased in gluconeogenic conditions and may be implicated in the development of diabetes. Higher rates of gluconeogenesis in PHG may be explained by a failure of insulin to inhibit gluconeogenesis in the setting of decreased insulin sensitivity [93]. Indeed, as revealed by QUICKI and HOMA-IR index, PHG had reduced insulin sensitivity with a more pronounced effect in male offspring. In line with this observation, notable sex-specific differences with regard to glucose metabolism were reported and females were shown to have higher whole-body insulin sensitivity than males [94]. The exact mechanism responsible for sex-specific differences in insulin sensitivity is not well understood, however, sex hormones or adipokines were proposed as potential contributors [95]. TAT which catalyzes the conversion of tyrosine to 4-hydroxyphenylpyruvate was another transaminase elevated in the PHG liver. In line, metabolomics showed near significance of reduced levels of tyrosine (l2fc = −0.47, adjusted p-value = 0.06), suggesting an active tyrosine catabolism. TAT is a gluconeogenic enzyme which is activated in the liver shortly after birth [96]. A potential mediator of decreased insulin sensitivity in PHG might be elevated NEFA levels [97]. Even slight elevation in plasma NEFA, whose flux is high, can significantly increase hepatic uptake [98]. Interestingly, higher expression of interferon-stimulated genes (ISGs) was observed in insulin resistant human patients [99]. ISG15 was positively correlated with insulin sensitivity and glucose homeostasis in humans and mice [100]. ISGs are a group of genes that are stimulated in response to interferon, thus their upregulation may hint towards inflammation due to an immune response [99]. Low-grade chronic inflammation may be a potential driver of insulin resistance in obesity and NAFLD [101]. A recent study reported the enrichment of ISGs, including IFI44, in GDM human amniocytes [102]. Besides, the metabolic-inflammatory circuit that links perturbations in lipid homeostasis with the activation of innate immunity was suggested [103]. Taken together, upregulation of gluconeogenic precursors and related enzymes suggests higher rates of gluconeogenesis in PHG liver which may be associated with impaired insulin sensitivity and glucose intolerance in later life. In conclusion, using a clinically relevant large animal model we showed that maternal hyperglycemia without confounding obesity results in profound metabolic alterations in the neonatal offspring's liver. Specifically, maternal hyperglycemia was related with increased rates of hepatic gluconeogenesis, amino acid metabolism and beta-oxidation but decreased rates of lipogenesis in PHG. Additionally, we found that hepatic PC biosynthesis was reduced while catabolism and translocation to bile was increased in PHG. We hypothesize that elevated PC levels despite reduced biosynthesis may be due to increased transplacental transfer and subsequent downregulation of enzymes involved in its synthesis via a feedback loop mechanism. In this study protein abundance changes alongside with quantitative data of metabolites were used as a proxy for the state of biochemical processes, however, our comprehensive dataset would greatly benefit from future studies assessing further measures of protein activity such as protein interactions and post-translational modifications. The generated datasets provide an important resource for future comparative or meta-analysis studies on the progression of hepatic complications and other associated comorbidities in neonatal offspring due to isolated maternal hyperglycemia.

## Funding

This study was supported by the German Center for Diabetes Research (DZD e.V.). This project has received funding from the European Union’s Horizon 2020 research and innovation programme under the Marie Skłodowska-Curie grant agreement No 812660 (DohART-NET).

## Author contributions

B.S., L.V., E.W., E.K. and T.F.: Conceptualization; B.S.: Methodology, Software, Data curation and analysis, Writing- Original draft; B.S., L.V., S.D.L., C.P., M.H., F.R., R.E., B.R., J.M. and M.H.d.A.: Investigation; L.V., S.D.L., J.B.S., S.R., E.W., E.K. and T.F.: Writing- Reviewing and Editing; E.W., E.K. and T.F.: Supervision, Resources, Funding acquisition

## Data and resource availability

The mass spectrometry proteomics data generated and analyzed in this study have been deposited to the ProteomeXchange Consortium via the PRIDE [45] partner repository, http://proteomecentral.proteomexchange.org; PXD040305. The metabolomics and clinical parameters results are included in the article/Supplementary Materials. Code to reproduce statistical analysis and visualization is available at: https://github.com/bshashikadze/maternaldiabetes-offspring-liver-omics-paper

## Declaration of Competing Interest

The authors declare that they have no known competing financial interests or personal relationships that could have appeared to influence the work reported in this paper.

## Data Availability

Datasets and codes used for the analysis and visualization have been deposited in dedicated repositories and the links are included in the manuscript
