# Supplementary material for: Maternal hyperglycemia induces alterations in hepatic amino acid, glucose and lipid metabolism of neonatal offspring: Multi-omics insights from a diabetic pig model
Source: Mol Metab. 2023 Jul 4;75:101768. doi: 10.1016/j.molmet.2023.101768 (PMC10372374; doi:10.1016/j.molmet.2023.101768)
Supplement: Multimedia component 1 [file mmc1.pdf]

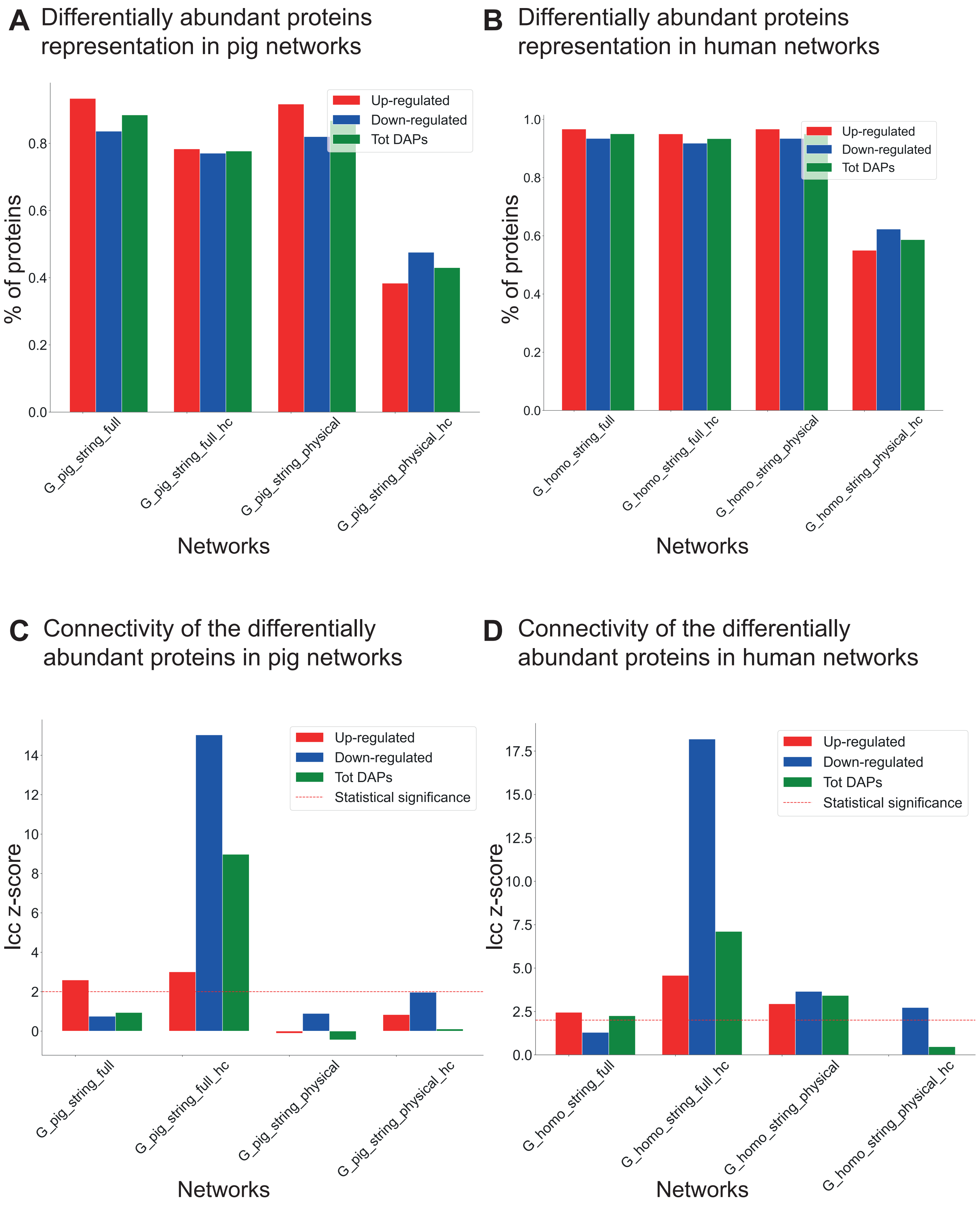
A
Differentially abundant proteins representation in pig networks
B
Differentially abundant proteins representation in human networks
C
Connectivity of the differentially abundant proteins in pig networks
D
Connectivity of the differentially abundant proteins in human networks
% of proteins
lcc z-score
Networks
Up-regulated
Down-regulated
Tot DAPs
Statistical significance
G_pig_string_full
G_pig_string_full_hc
G_pig_string_physical
G_pig_string_physical_hc
G_homo_string_full
G_homo_string_full_hc
G_homo_string_physical
G_homo_string_physical_hc

Supplementary Figure 1 Comparison of different network characteristics for different STRING-derived networks. **A**: Percentage of differentially abundant proteins in different STRING-derived pig-specific PPI (full, full with high confidence, physical, physical with high confidence). **B**: Percentage of differentially abundant proteins in different STRING-derived human-specific PPI (full, full with high confidence, physical, physical with high confidence). **C**: Connectivity among the differentially abundant proteins in different STRING-derived pig-specific PPI (full, full with high confidence, physical, physical with high confidence). **D**: Connectivity among the differentially abundant proteins in different STRING-derived human-specific PPI (full, full with high confidence, physical, physical with high confidence).

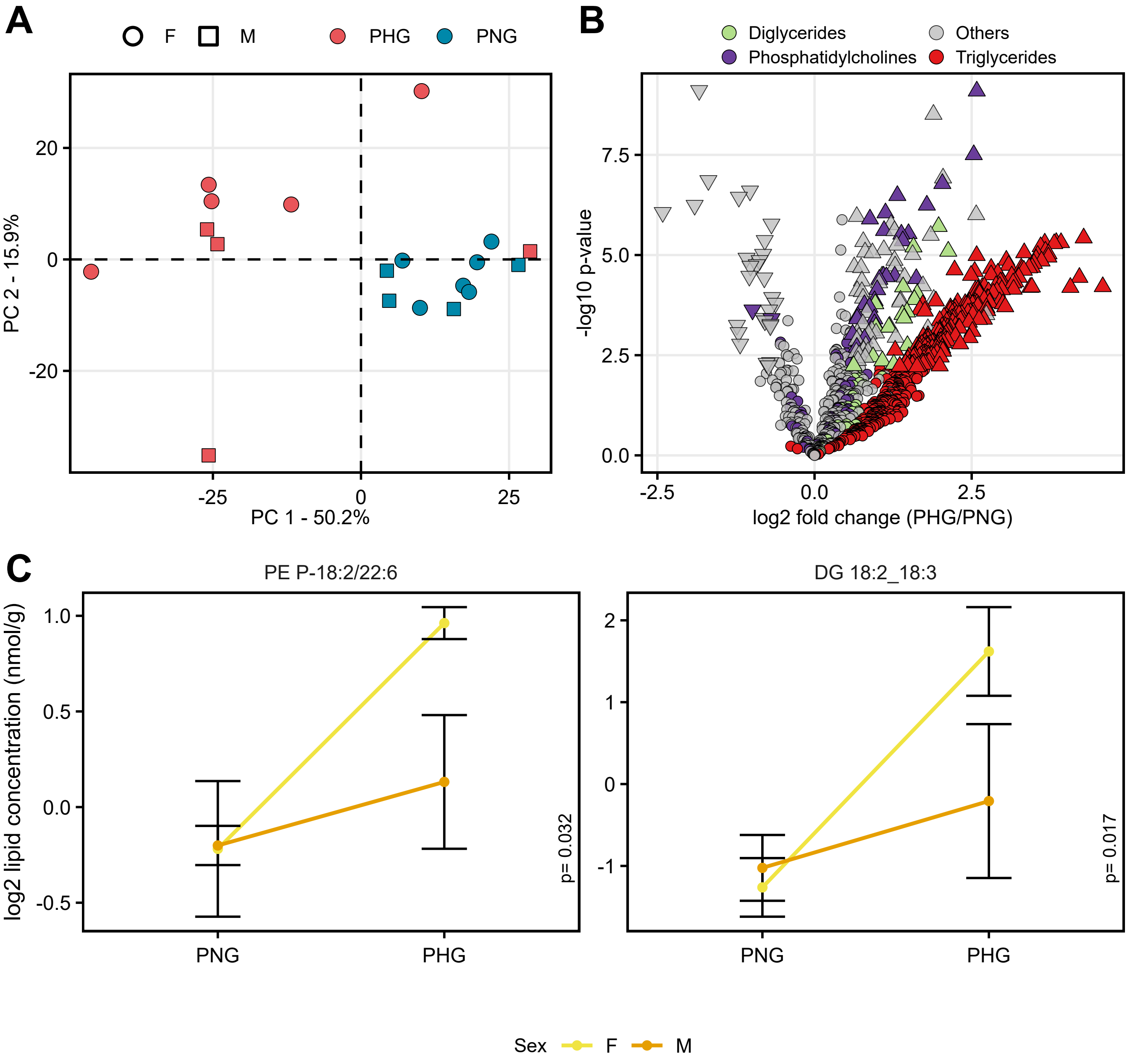
A
F
M
PHG
PNG
PC 2 - 15.9%
PC 1 - 50.2%
20
0
-20
-25
0
25
B
Diglycerides
Phosphatidylcholines
Others
Triglycerides
-log10 p-value
log2 fold change (PHG/PNG)
7.5
5.0
2.5
0.0
-2.5
0.0
2.5
C
PE P-18:2/22:6
DG 18:2_18:3
log2 lipid concentration (nmol/g)
1.0
0.5
0.0
-0.5
2
1
0
-1
PNG
PHG
p= 0.032
p= 0.017
Sex
F
M

Supplementary Figure 2 Overview of shotgun lipidomic analysis of the liver from hyperglycemia exposed and control offspring. **A:** Principal component analysis of log2 transformed and unit variance scaled data. The shape of each spot corresponds to the sex, and the color to the mother's genotype. **B:** Volcano plot comparing the lipid abundance change between conditions (PHG/PNG). Significantly changed lipids (Benjamini-Hochberg adjusted p-value ≤ 0.05 and fold change ≥ 1.5) are shown as up- and down-pointing triangles for increased and decreased abundance in PHG versus PNG, respectively. Circles correspond to non-significant changes. The x and y axis show the log2 fold-change in metabolite levels and the log10 two-way ANOVA group p-value, respectively. Selected lipid species are color-coded. **C:** Lipids with the significant group*sex interaction effect (Benjamini-Hochberg adjusted p-value ≤ 0.05) from the two-way ANOVA. Error bars correspond to the standard deviation.

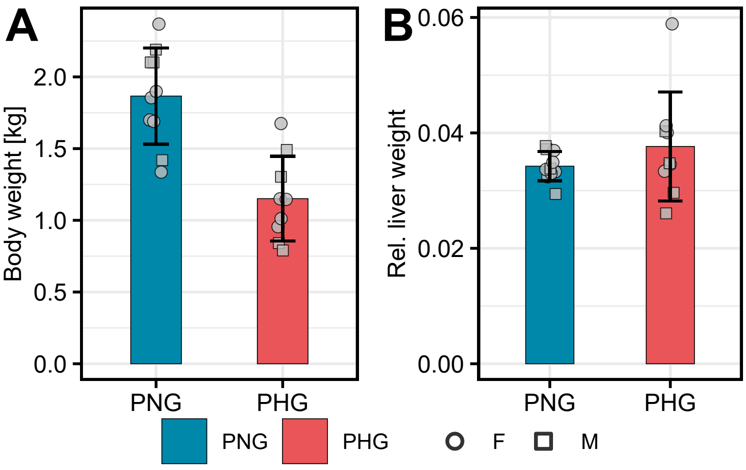
A
Body weight [kg]
2.0
1.5
1.0
0.5
0.0
PNG
PHG
B
Rel. liver weight
0.06
0.04
0.02
0.00
PNG
PHG
PNG
PHG
F
M

Supplementary Figure 3 (A) body weight and (B) liver weight relative to the body weight. P-values were 0.0003, 0.82 and 0.47 for the effect group, sex and interaction group*sex, respectively for the body weight. P-values were 0.26, 0.19 and 0.13 for the effect group, sex and interaction group*sex, respectively for the relative liver weight. Error bars correspond to the standard deviation.

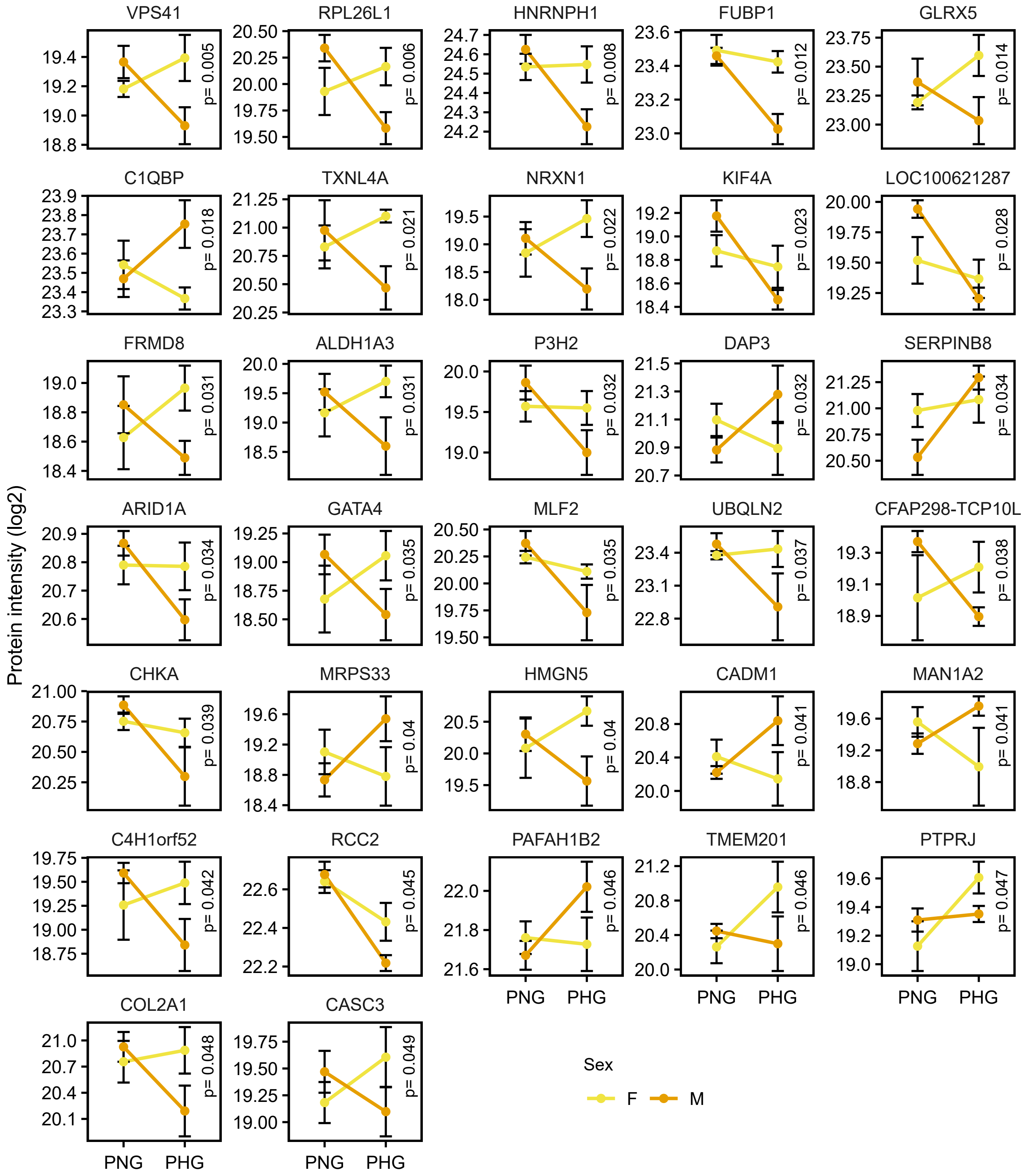

Protein intensity (log2)
VPS41
p= 0.005
RPL26L1
p= 0.006
HNRNPH1
p= 0.008
FUBP1
p= 0.012
GLRX5
p= 0.014
C1QBP
p= 0.018
TXNL4A
p= 0.021
NRXN1
p= 0.022
KIF4A
p= 0.023
LOC100621287
p= 0.028
FRMD8
p= 0.031
ALDH1A3
p= 0.031
P3H2
p= 0.032
DAP3
p= 0.032
SERPINB8
p= 0.034
ARID1A
p= 0.034
GATA4
p= 0.035
MLF2
p= 0.035
UBQLN2
p= 0.037
CFAP298-TCP10L
p= 0.038
CHKA
p= 0.039
MRPS33
p= 0.04
HMGN5
p= 0.04
CADM1
p= 0.041
MAN1A2
p= 0.041
C4H1orf52
p= 0.042
RCC2
p= 0.045
PAFAH1B2
p= 0.046
TMEM201
p= 0.046
PTPRJ
p= 0.047
COL2A1
p= 0.048
CASC3
p= 0.049
PNG
PHG
Sex
F
M

Supplementary Figure 4 Proteins with the significant group*sex interaction effects (Benjamini-Hochberg adjusted p-value ≤ 0.05) from the two-way ANOVA. Error bars correspond to the standard deviation.

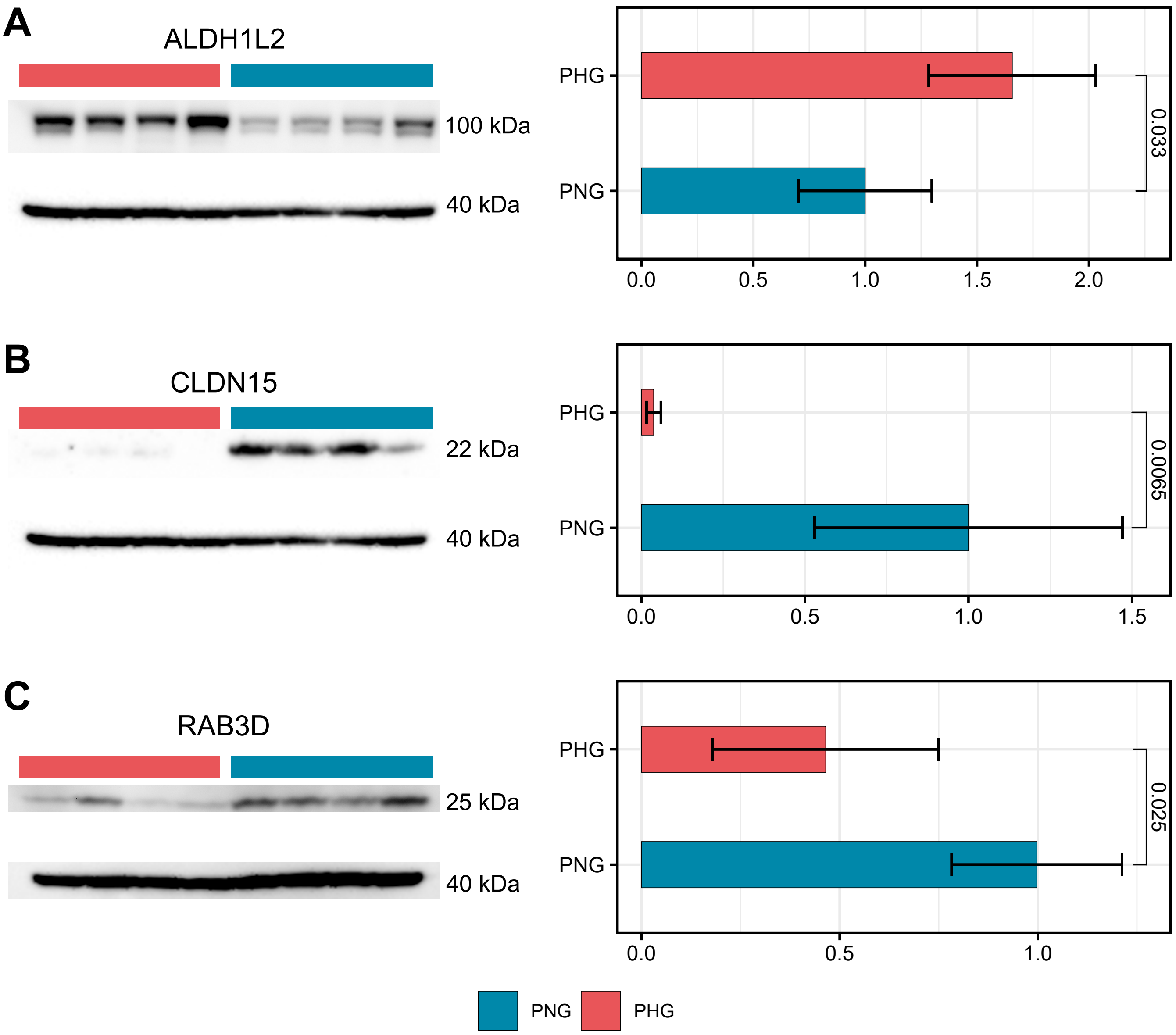
A
ALDH1L2
100 kDa
40 kDa
PHG
PNG
0.033
0.0
0.5
1.0
1.5
2.0
B
CLDN15
22 kDa
40 kDa
PHG
PNG
0.0065
0.0
0.5
1.0
1.5
C
RAB3D
25 kDa
40 kDa
PHG
PNG
0.025
0.0
0.5
1.0
PNG
PHG

Supplementary Figure 5 Western blot analysis of selected protein candidates confirms mass spectrometry-based quantitative data. Significance was assessed using Student's *t*-test. Actin was used as a loading control (bottom Western blots).

A Downregulated proteins core connectivity

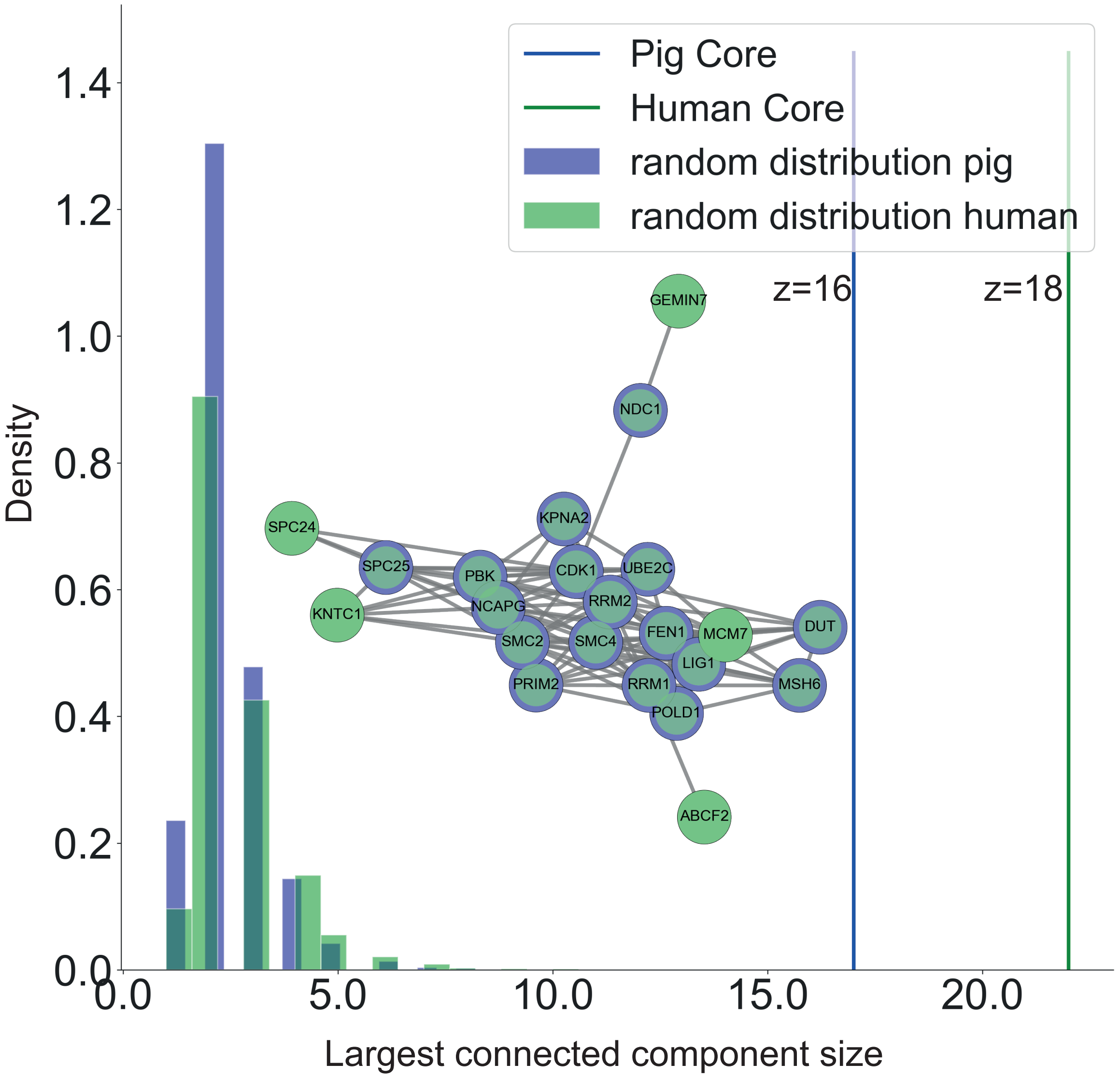

B Downregulated proteins module

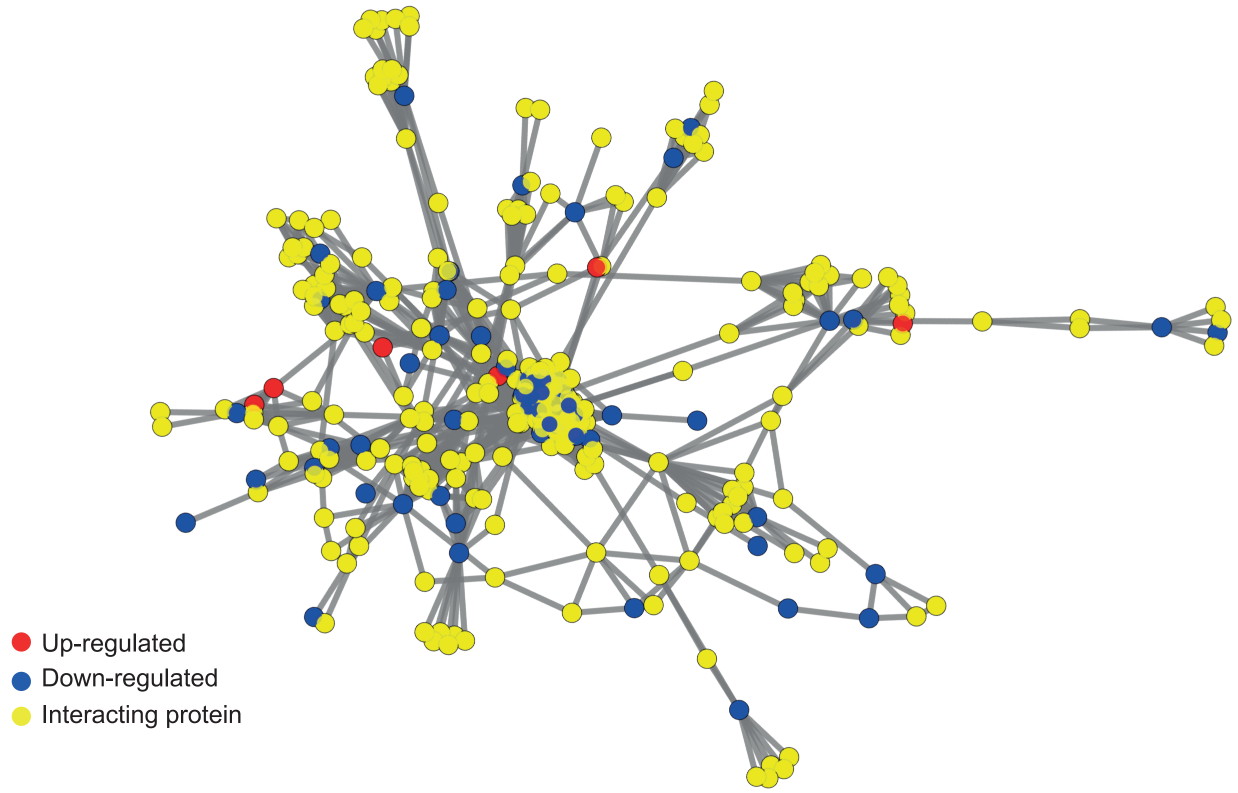

Supplementary Figure 6 Network analysis of down-regulated proteins. **A:** Identification of the down-regulated network core that deviates from random expectation (10,000 random sets of proteins of equal size) based on its connectivity ($z\text{-score}_{pig}$=16, $z\text{-score}_{human}$=18). The green color refers to the human PPI, and the blue color to the pig PPI. **B:** Down-regulated expanded network which contains all down-regulated proteins and their interacting partners. This subnetwork is formed by 363 proteins colored in red if up-regulated in PHG, blue if down-regulated in PHG, and yellow if not detected but interacting with differentially abundant proteins.

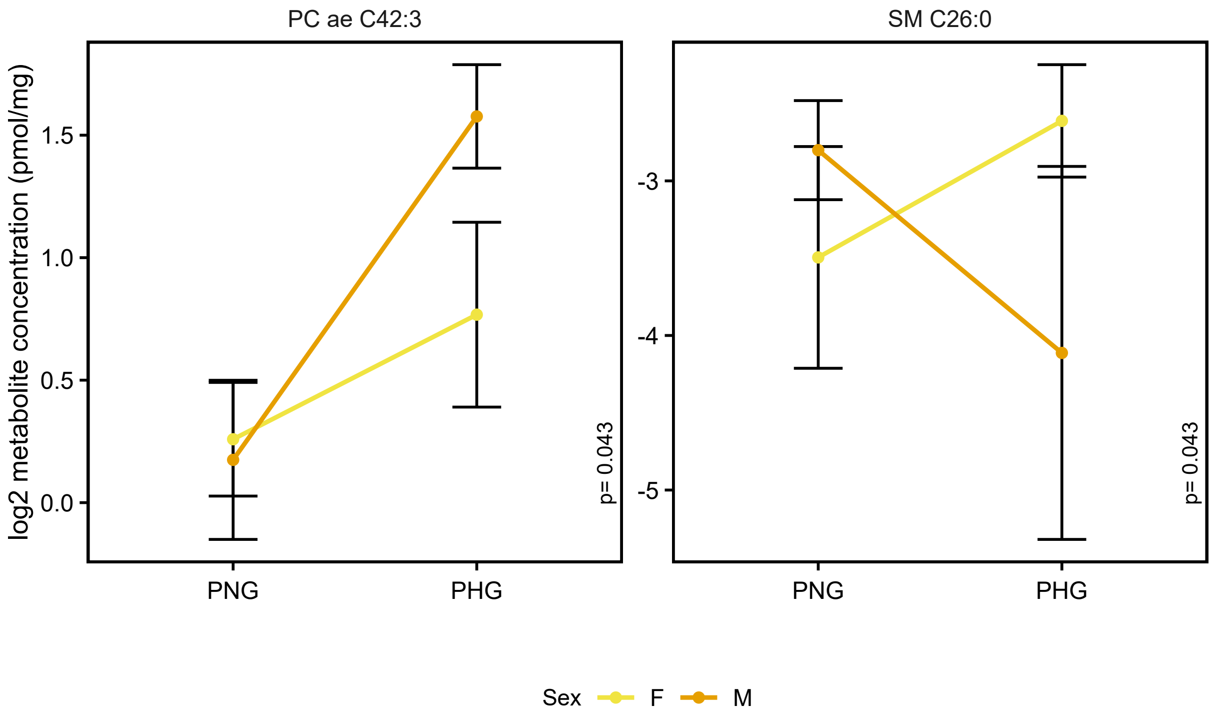

PC ae C42:3
SM C26:0
log2 metabolite concentration (pmol/mg)
1.5
1.0
0.5
0.0
-3
-4
-5
p= 0.043
p= 0.043
PNG
PHG
PNG
PHG
Sex
F
M

Supplementary Figure 7 Metabolites with the significant group*sex interaction effects (Benjamini-Hochberg adjusted p-value ≤ 0.05) from the two-way ANOVA. Error bars correspond to the standard deviation.

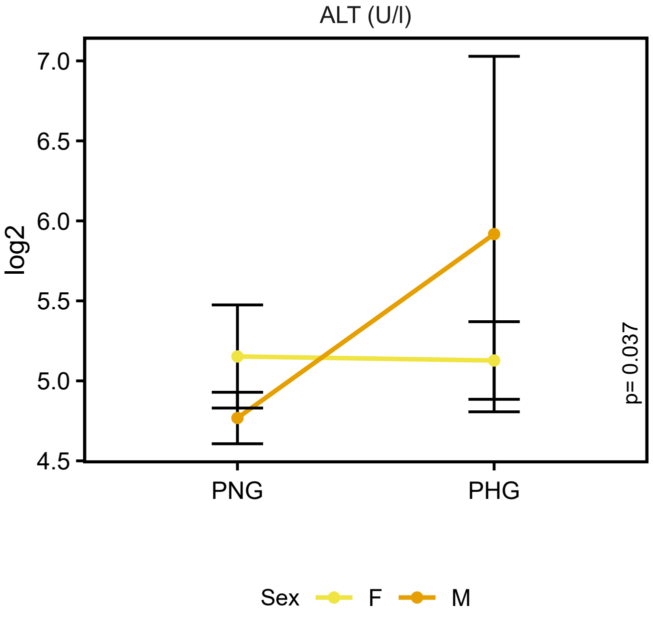
ALT (U/l)
7.0
6.5
6.0
5.5
5.0
4.5
log2
p= 0.037
PNG
PHG
Sex
F
M

Supplementary Figure 8 Clinical chemical parameter with the significant group*sex interaction effect (Benjamini-Hochberg adjusted p-value ≤ 0.05) from the two-way ANOVA. Error bars correspond to the standard deviation.
